# The R package *otu2ot* for implementing the entropy decomposition of nucleotide variation in sequence data

**DOI:** 10.3389/fmicb.2014.00601

**Published:** 2014-11-14

**Authors:** Alban Ramette, Pier Luigi Buttigieg

**Affiliations:** ^1^HGF-MPG Group for Deep Sea Ecology and Technology, Max Planck Institute for Marine MicrobiologyBremen, Germany; ^2^Organic Geochemistry Department, MARUM - Center for Marine Environmental SciencesBremen, Germany; ^3^HGF-MPG Group for Deep Sea Ecology and Technology, Alfred-Wegener-Institut Helmholtz-Zentrum für Polar- und MeeresforschungBremerhaven, Germany

**Keywords:** oligotyping, minimum entropy decomposition, one-pass decomposition, diversity, next generation sequencing

## Abstract

Oligotyping is a novel, supervised computational method that classifies closely related sequences into “oligotypes” (OTs) based on subtle nucleotide variation (Eren et al., 2013). Its application to microbial datasets has helped reveal ecological patterns which are often hidden by the way sequence data are currently clustered to define operational taxonomic units (OTUs). Here, we implemented the OT entropy decomposition procedure and its unsupervised version, Minimal Entropy Decomposition (MED; Eren et al., 2014c), in the statistical programming language and environment, R. The aim of this implementation is to facilitate the integration of computational routines, interactive statistical analyses, and visualization into a single framework. In addition, two complementary approaches are implemented: (1) An analytical method (the broken stick model) is proposed to help identify OTs of low abundance that could be generated by chance alone and (2) a one-pass profiling (OP) method, to efficiently identify those OTUs whose subsequent oligotyping would be most promising to be undertaken. These enhancements are especially useful for large datasets, where a manual screening of entropy analysis results and the creation of a full set of OTs may not be feasible. The package and procedures are illustrated by several tutorials and examples.

## Introduction

Eren et al. (2013) implemented a technique called *oligotyping* to help identify highly variable nucleotide positions of 16S rRNA gene sequences by calculating their Shannon entropy values. Subtle variations are used to iteratively classify the sequences into oligotypes (OTs), which may offer an interesting way to resolve ecologically meaningful differences between closely related organisms. In some cases, especially when processing data generated from sequencing methods prone to insertions or deletions (e.g. 454 Massively Parallel Tag Sequencing), sequence alignment must be performed prior to oligotyping to ensure meaningful classification (see the example below). The oligotyping procedure is straightforward: Sequences are assigned to the same taxonomic group or clustered together in one OTU before oligotyping analysis performs a systematic identification of nucleotide positions that represent information-rich variations across the group or OTU. The variation at these positions is then used to bin the sequences into OTs. If sample information is available for each sequence originating from one OTU, a sample-by-OT table is then produced, which can be subjected to traditional multivariate analyses (e.g., Legendre and Legendre, 1998; Ramette, 2007; Buttigieg and Ramette, in press).

Depending on the degree of variability in a sequenced region, the identity threshold between different OTs may be as low as 0.2%, i.e., about an order of magnitude lower than the 3% identity threshold that is currently being used to define OTUs. Consequently, the marginal diversity space left unexplored by coarse-grained methods requires attention and its significance needs to be assessed in its evolutionary and environmental context. Indeed, the subtle nucleotide variation detected by oligotyping among 16S ribosomal RNA gene amplicon reads has revealed ecologically meaningful microdiversity patterns hidden in sequence datasets. For instance, the technique has successfully identified subtle nucleotide variations that were associated with distinct environments, hosts, body location, or epidemiological states in human oral (Eren et al., 2014a), gut (Eren et al., 2014b), and bacterial vaginosis (Eren et al., 2011) microbiomes, but also in wastewater communities (McLellan et al., 2013), or among spatially structured communities in Arctic deep-sea sediments (Buttigieg and Ramette, submitted).

In addition to its ecological applications, the procedure is also computationally interesting because it identifies a relatively small subset of nucleotide positions in a set of sequences associated with high entropy values, thus reducing subsequent computational effort. However, the original oligotyping procedure is supervised: it relies on user input to decide how many components (i.e., positions with high entropy values) and which entropy threshold to be considered for further rounds of oligotyping. The supervised method may work when dealing with a few, well-targeted OTUs, but if we are to cope with very large datasets, as commonly encountered in environmental and clinical microbiology, a more scalable, automatic procedure is required. Recently, Eren and colleagues proposed a computationally efficient procedure to partition marker gene datasets in an unsupervised fashion, which they termed *Minimum Entropy Decomposition* (MED; http://oligotyping.org/MED/; Eren et al., 2014c). This approach iteratively partitions large sets of sequences by repeating the oligotyping procedure until no more high entropy nucleotide positions are identified in any of the partitions of those sequences.

With regard to their implementation, the original oligotyping and MED software scripts are written in Python to efficiently handle the FASTA sequences, Shannon entropy calculations, and navigation across numerous directories that are created during the successive rounds of OT generation. The following Python modules need to be manually installed: *Matplotlib* (http://matplotlib.sourceforge.net/), *BioPython* (http://biopython.org/wiki/Biopython), *SciPy* (http://www.scipy.org/), *PyCogent* (http://pycogent.org/), and *Django* (https://www.djangoproject.com/), to generate user-friendly HTML outputs. The final stage of data visualization and further ecological analysis of sample-by-OT patterns rely on using the R language (R Core Team, 2014) and its libraries. Several R scripts are used to reduce the dimensionality of large datasets, calculate dissimilarity matrices, or to visualize data (e.g., using the functions *heatmap* and *barplot*). The oligotyping and MED scripts also have some dependencies such as NCBI executable (especially *blastn*) to match the most interesting OT sequences directly to their closest relatives in local or publicly available sequence databases.

Here, the R package *otu2ot*, which stands for “OTU to OT” is described and examples as well as tutorials are provided to illustrate the library's installation and functioning. The oligotyping and MED routines are implemented solely using R scripts in order to facilitate the integration of computational routines, interactive statistical analyses, and visualization into one common framework. Additional methods are also presented such as the broken stick model procedure to help identify OTs of low abundance that could be generated by chance alone. Further, a one-pass entropy profiling approach is compared to MED, as a method to efficiently identify those OTUs whose decomposition into OTs would be most promising. This latter method is especially useful for large datasets, where a complete decomposition to OTs may not be computationally feasible.

## Methods

### R implementation and dependencies

R (http://www.R-project.org/) is a widely used language and environment for statistical computation and graphics. The core of R is an interpreted computer language which allows branching and looping as well as modular programming using functions. Although most of the user-visible functions in R are written in the R language itself, procedures written in the C, C++, or FORTRAN languages, can be easily called to further improve computational efficiency.

To develop *otu2ot*, R version 3.1.0 was used within RStudio (version 0.98.953; http://www.rstudio.com/). Within *otu2ot*, the R library *seqinR* (Charif and Lobry, 2007) is called to efficiently import FASTA sequences. The optional libraries *FactoMineR* (Husson et al., 2014) and *vegan* (Oksanen et al., 2013) may also be used to calculate specific coefficients and to perform multivariate analysis of community data, respectively, but are not mandatory to perform the oligotyping or MED procedures. The package can be easily installed as described in the tutorials (Supporting Information). An active repository is available at: https://github.com/aramette/otu2ot.

### Expected input data format

The *otu2ot* library expects input FASTA files to have a specific format, identical to that required by the original oligotyping pipeline, as described at: http://oligotyping.org/.

All of the (aligned) sequences from an OTU of interest have to be present in a single multi-FASTA file, and all reads must have the following format:

>[SampleName]_[ReadId]

GTTGAAAAAGTTAGTGGTGAAATCCCAGA

where “[SampleName]” refers to the name of the sample from which the sequences originated from and “[ReadId]” refers to a unique sequence identifier.

### Differences to original oligotyping and MED implementations

In its current version (1.4), *otu2ot* does not implement two optional features found in the original procedure: (1) the selection of several components in the MED procedure, and (2) the subsequent BLAST analysis of the most abundant unique OT sequences against NCBI's *nr* database. This latter option may be readily integrated using additional R libraries such as BoSSA (http://cran.r-project.org/web/packages/BoSSA/) at a later stage. Other features are implemented, however, namely the broken stick model (BSM) and a one-pass (OP) procedure, as follows.

The BSM is implemented to help identify which OTs have a read abundance greater than one would expect by chance. Following the decomposition of an OTU into OTs, only those OTs which satisfy this condition are further considered for community analysis. The original BSM idea originates from niche theory (MacArthur, 1957), where the sub-division of niche space among species is thought to be analogous to randomly breaking a stick into *p* pieces. When applied to oligotyping data, the procedure is as follows: The total number of sequences clustered into one OTU is randomly split into *p* subsets (i.e., “pieces” of the broken stick) where *p* is defined by the number of OTs detected. The pieces are then sorted by decreasing size. By repeating these two steps many times and averaging the results over all executions the BSM generates the OT abundances which would occur by chance alone, that is, the distribution of OT abundances if there was no structure in the data. The R script used in our implementation uses a simple formula that provides the expected abundance values for a given partition under the BSM (Legendre and Legendre, 1998):

$$b_{k}=\frac{1}{p}\sum_{i=k}^{p}\frac{1}{i}$$

where *p* is the number of pieces (i.e., the number of OTs) and *b_k_* is the expected abundance of the kth OT under the BSM.

One may then choose to limit their analyses to those OTs whose abundances are larger than those generated by the BSM. This procedure allows the use of a null abundance model to focus on OTs whose abundances are likely to be non-random, instead of relying on an arbitrary choice of minimum OT abundance or on external knowledge to allow a given OT to be further considered for downstream analyses. This approach may thus help lessen the subjectivity which threatens reproducibility and consistency in defining what a minimum OT abundance should be. The BSM has been advocated as appropriate to describe the right-hand side of the rank frequency curve, i.e., the distribution of the rare species (Frontier, 1985), so it may be useful for OT abundance distributions, which are conceptually similar. In addition, the same approach is often applied to the solution of a principal component analysis in order to suggest the minimum number of principal axes needed to satisfactorily represent a data matrix (Legendre and Legendre, 1998). However, when considering results from oligotyping procedures, it is important to note that other models of species distribution exist and should also be evaluated (e.g., the log series, log normal, or neutral model): it would be hasty to favor the BSM over any alternatives at this stage. Future research using, for instance, simulated datasets with known amounts of sequencing error or rare sequences could be used to validate the application of the BSM approach to OT abundance modeling.

A one-pass (OP) procedure is also proposed to rapidly assess the amount of microdiversity present in a set of sequences. The procedure is similar to oligotyping, but it only performs one round of entropy calculations. When an entropy profile is obtained, only the nucleotide positions with Shannon entropy values greater than a chosen threshold are concatenated, and these concatenated sequences are then used to classify the sequences into OTs. Here we determined how OP compares to MED, in terms of computational speed and in its ability to capture ecological information such as variance in community composition, community patterns, or presence of rare types (e.g., singletons). We also evaluated whether OP can be used as a first screen across a large number of OTUs, before using the more computationally-demanding MED procedure to analyze microbial diversity on targeted OTUs.

### Future developments

The motivation behind the creation of *otu2ot* is twofold. First, it provides a more transparent, single-language implementation of the scripts used for oligotyping and MED, in order to promote more development of these tools and approaches. At this stage, less emphasis has been given to the improvement of computational performance, but this could be obtained by code optimization and interfacing with C or C++. This should be addressed when the phase of prototyping methods such as oligotyping, MED, or OP is over and large datasets need to be efficiently analyzed. In that respect, R is receiving much attention and is being actively developed to support very efficient parallel computing solutions, large memory data handling, and seamless interfacing with compiled code (e.g., http://cran.r-project.org/web/views/HighPerformanceComputing.html). R also has a large and growing user base among data scientists and ecologists, who continually submit new packages which can be integrated with *otu2ot*, further motivating development in this language. To improve interactive data exploration and visualization, developers have contributed R libraries such as *shiny* (http://shiny.rstudio.com/), which may readily turn a set of R functions into interactive web interfaces. Beyond R and interfacing with C or C++, other high-level languages may also be used to efficiently implement oligotyping, MED, or OP, at least for the entropy decomposition steps. These may be worth comparing to this R implementation in the future. For instance, the Julia language (http://julialang.org/) is a high-performance, dynamic programming language with a syntax that would be familiar to R users, and which seems to improve computing speed by several orders of magnitude when compared to a range of functions implemented in R.

### Example datasets

The original data available (e.g., “mock” dataset) on the website (http://oligotyping.org/) were used to create the R functions and ensure that results were concordant with those of the original implementation. These data are also included in the *otu2ot* package. The dataset used in Buttigieg and Ramette's (submitted) application of oligotyping was also used here. It corresponds to a set of sequence-abundant OTUs (abundance greater than or equal to 100 reads), derived from sequencing of sediment samples from the Hausgarten Long-Term Ecological Research station (Eastern Fram Strait, Arctic sea), which were clustered at the 97% sequence identity level of the 16S rRNA gene. The sequence data were produced by 454 Massively Parallel Tag Sequencing, and sequence alignment was performed to account for insertions and deletions. The analysis of the full OTU dataset from this site was previously published (Jacob et al., 2013). All relevant datasets are provided as Supplementary Information.

In the following section, a few plots and results are provided to illustrate how to use the *otu2ot* package and its functions. Here, we compare the results and performance of different methods and less emphasis is given to the ecological interpretation of the resulting OT tables, which can be found elsewhere (Buttigieg and Ramette, submitted). It should be noted that our examples began at an OTU-level resolution and further explored the extent of OT microdiversity within OTUs. It is equally interesting to choose a coarser taxonomic level (e.g., Phylum, Class) where more robust membership is expected, and then perform oligotyping methods. This would alleviate issues originating from splitting sequences into different OTUs as a result of the OTU clustering step.

## Examples of application

### MED analysis of one OTU dataset

**Figure 1:**
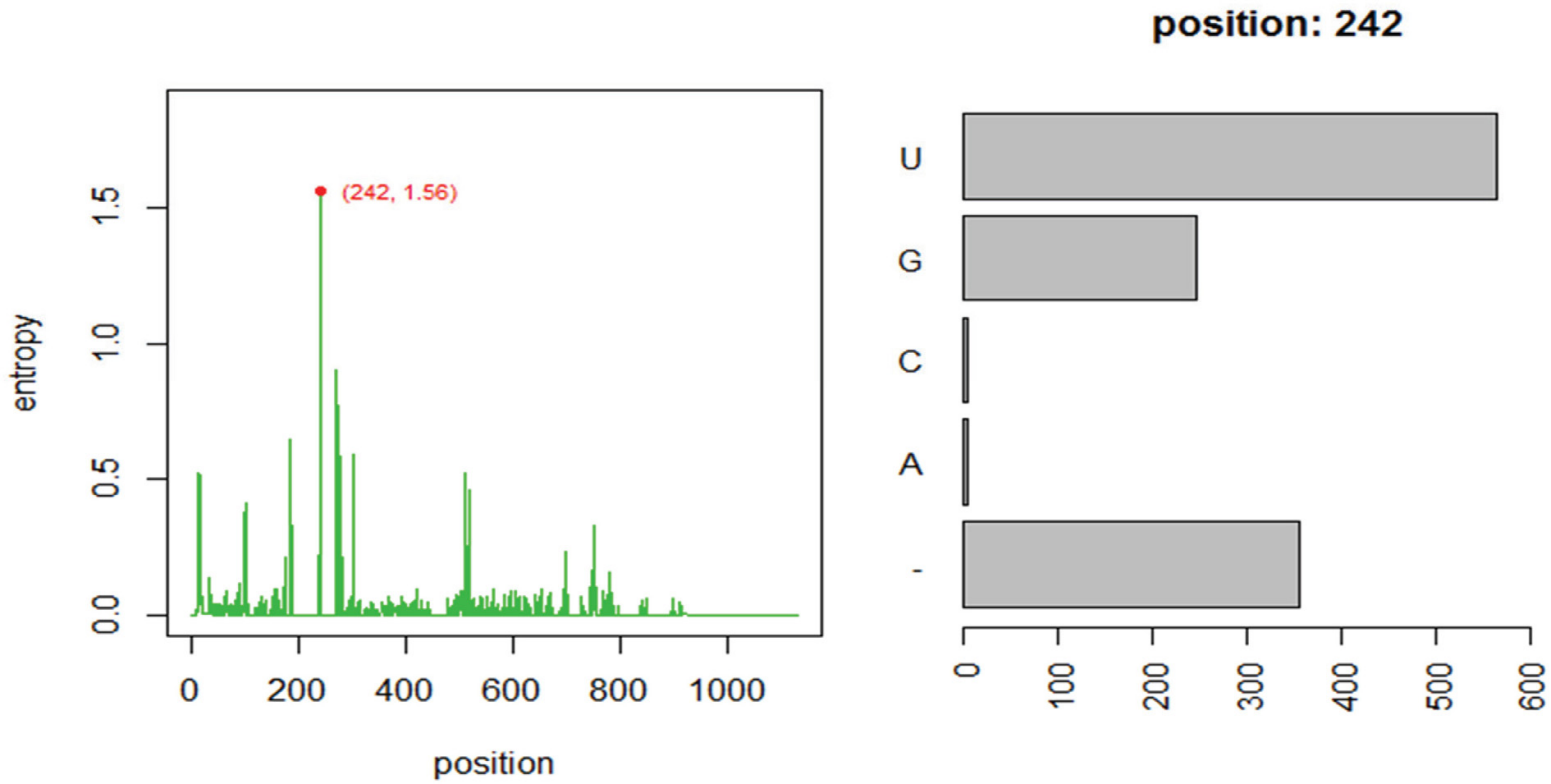
**Entropy profile of file “HGB_0013_GXJPMPL01A3OQX.fasta” and further nucleotide composition of the position of higher Shannon entropy (position 242)**.

**Figure 2:**
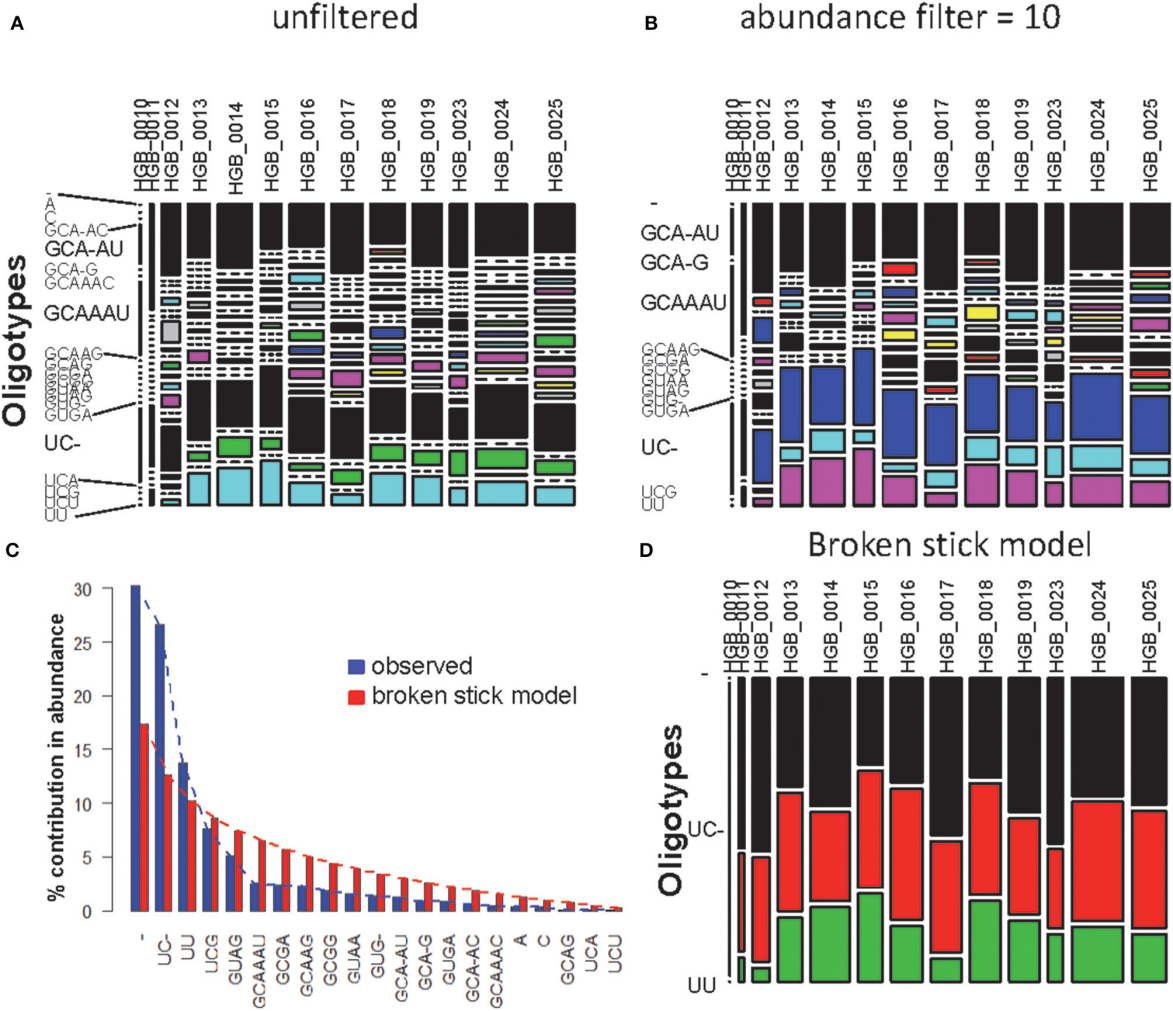
**MED analysis of HGB_0013_GXJPMPL01A3OQX.fasta. (A)** Raw compositional table, **(B)** filtered by minimum OT abundance of 10, **(C)** Comparison of observed OT abundance vs. expected under the BSM, and **(D)** compositional table filtered by BSM.

Using one abundant OTU (1175 sequences, 1133 positions) whose sequences are provided in file HGB_0013_GXJPMPL01A3OQX.fasta, we generated a Shannon entropy profile of the alignment and a nucleotide composition profile of the position with the highest Shannon entropy (position 242). Note that alignment gaps (−) are also considered as informative in these calculations (Figure 1; Tutorial 1). By using the sample information in each sequence header, a raw sample-by-OT compositional table was generated (Figure 2A), which can be filtered by minimum OT abundance in the table (Figure 2B) or further filtered by applying the broken stick model (BSM) rule (Figures 2C,D).

### One-pass (OP) approach

**Figure 3:**
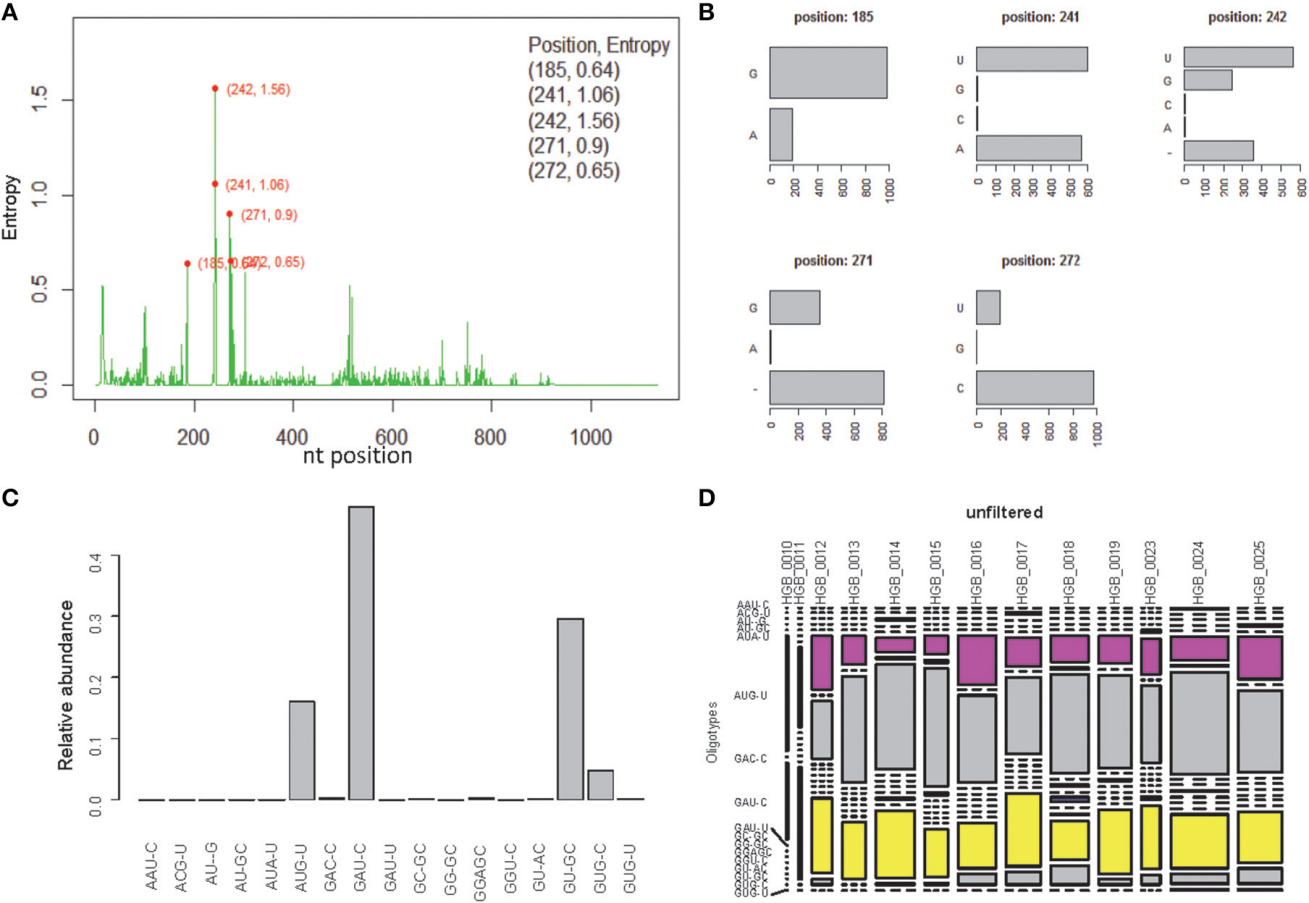
**One-Pass (OP) analysis of HGB_0013_GXJPMPL01A3OQX.fasta. (A)** Shannon entropy profile, **(B)** nucleotide composition of the 5 high-entropy positions, **(C)** Relative abundance of each OT obtained by OP, **(D)** raw compositional table.

**Figure 4:**
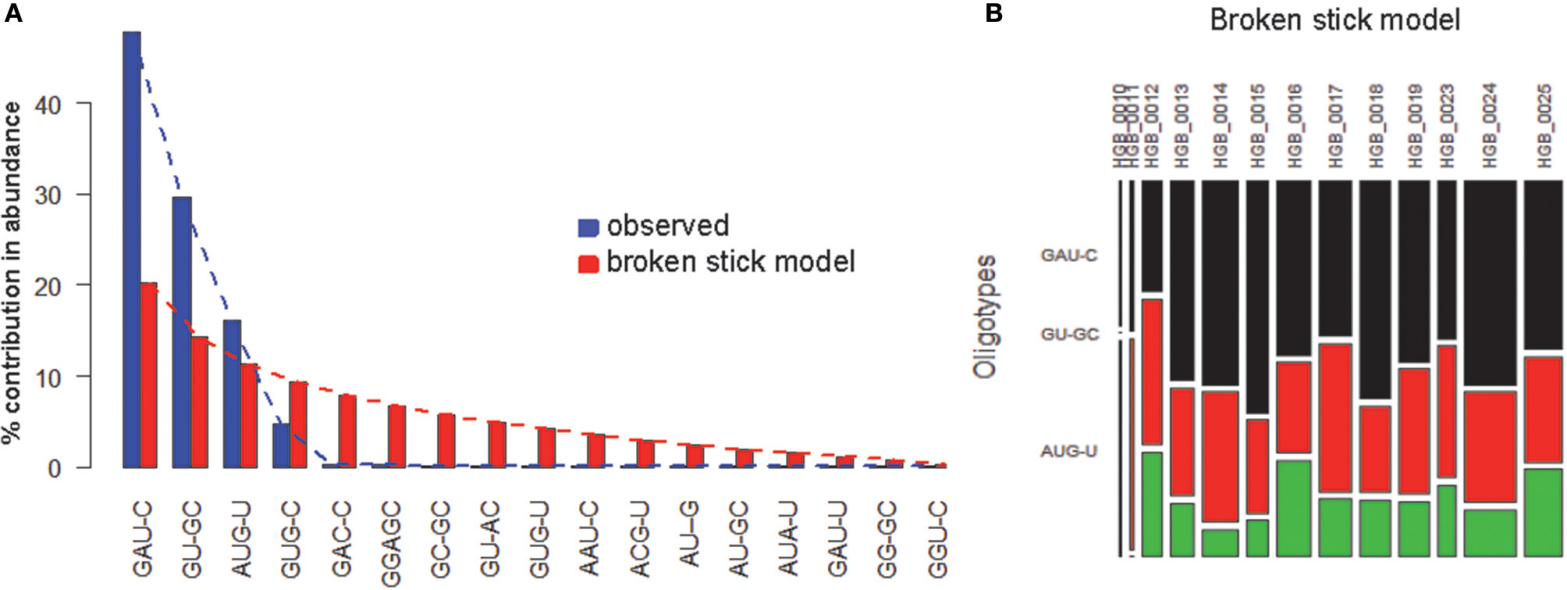
**BSM filtering applied to the OT table generated from HGB_0013_GXJPMPL01A3OQX.fasta by OP. (A)** broken-stick model evaluation, **(B)** BSM filtered compositional table.

OP analysis of the same alignment file indicated 5 positions associated with high Shannon entropy values (Figures 3A,B; Tutorial 2). Further concatenation and binning of the sequence data led to 4 dominant OTs (Figure 3C) out of the 17 OTs generated by OP. Most of the rarer OTs were, in fact, singletons (Figure 3D). Subsequent BSM filtering (Figure 4A) led to a compositional table (Figure 4B) very similar to the one obtained by MED followed by BSM filtering (Figure 2D). Despite those similar plots, a number of differences may be observed which require careful investigation to fully compare the results produced by OP and MED (Tutorial 3).

As expected, the OP table has fewer columns (corresponding to 17 OTs) than the MED table (21 OTs). MED splits the initial number of sequences (1175) to greater extent, but OP displays more singleton OTs. When OT abundances were correlated across tables, high correlation values were mostly obtained among abundant OTs (Table 1), particularly for OT abundances >50 sequences. This may explain why community patterns that are extracted by multivariate techniques, many of which focus on the most abundant types, were found to be very similar overall. Because OP does not decompose the sequence pool to the same extent as MED, many OTs obtained by MED (11 out of 21), including some rather abundant ones, did not correlate with any OTs obtained by OP.

**Table 1:**
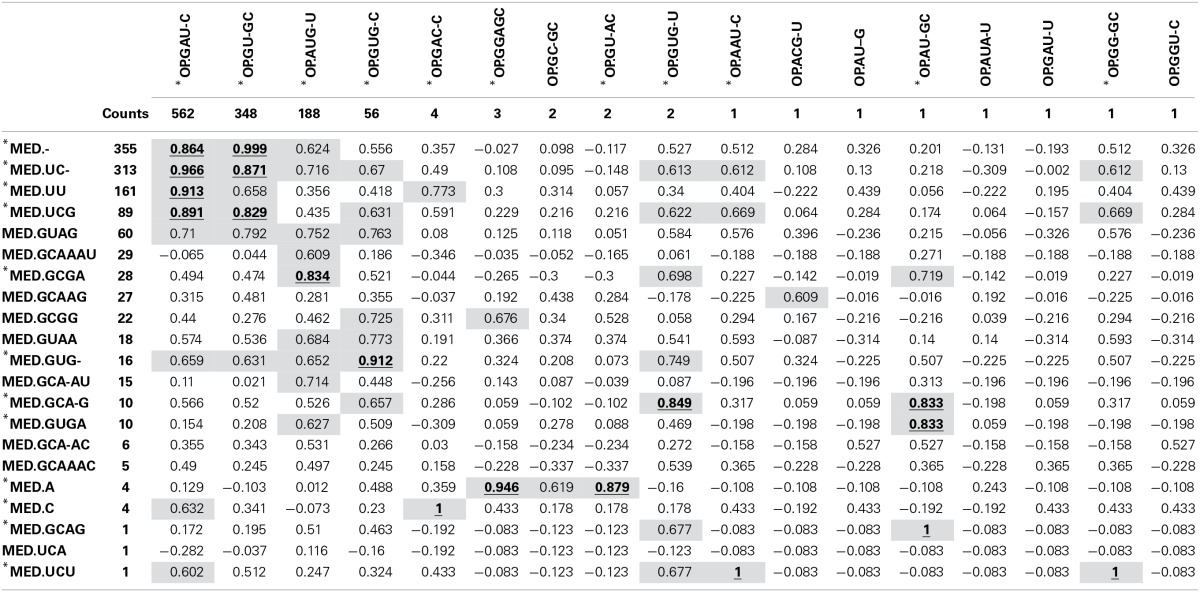
**Correlation between OT abundance values obtained by MED (rows) and those obtained by OP (columns)**.

Pearson correlation coefficients > 0.6 (absolute values) are indicated in gray. Bold, underlined values are those >0.8. OT names with an asterisk (^*^) are those which are associated with a correlation coefficient > 0.8 at least once, indicating a good match between the two methods.

**Figure 5:**
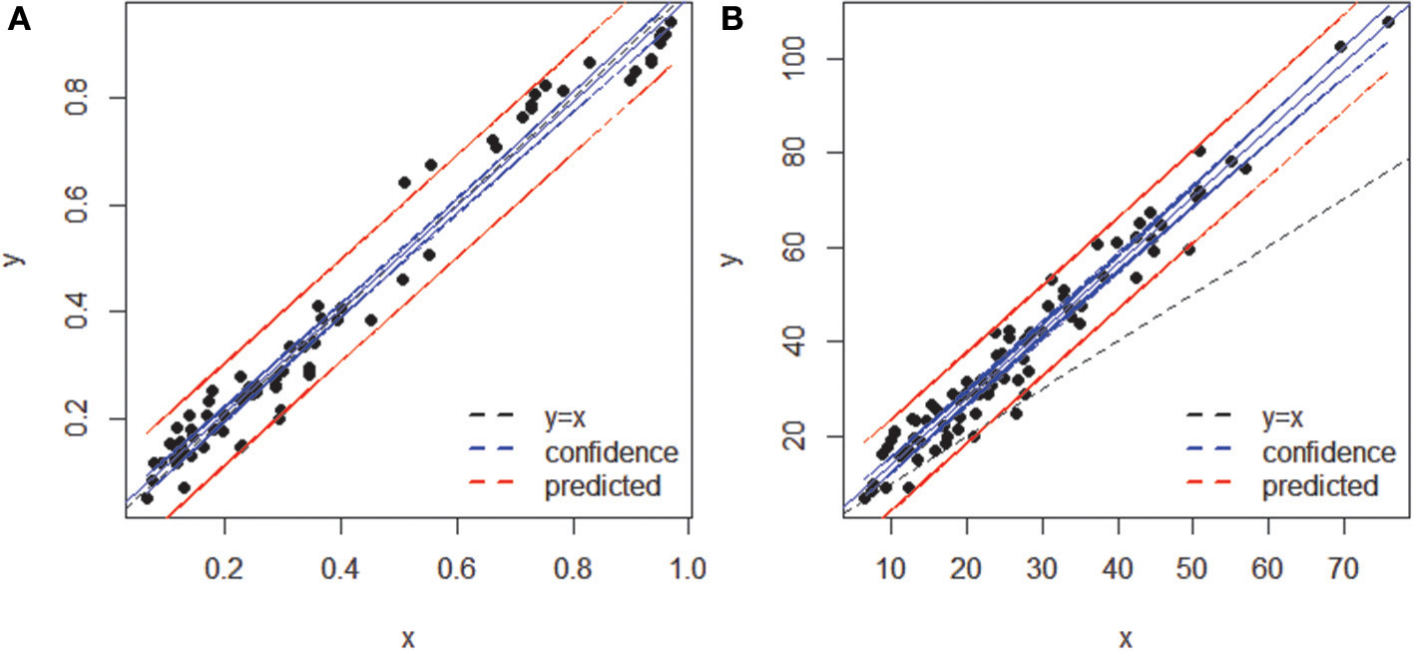
**Comparison of sample dissimilarities obtained by MED (y axis) and of those obtained by OP (x axis) **(A)** using an asymmetric (Bray-Curtis) dissimilarity coefficient, **(B)** using a symmetric (Euclidean) coefficient**. Notice the departure from the 1:1 line due to the inclusion of rare OTs in (B). The blue and red lines represent confidence (95%) and prediction lines, respectively, of the linear regression models.

When both OT tables were rarefied according to the BSM, both were left with only 3 OTs, corresponding to 70.5 and 93.4% of all sequences for MED and OP, respectively, and which led to very similar abundance profiles (Table 2). Interestingly, the total community variance still present in each dataset was very different with nearly twice as much for OP (953) as for MED (472) (Table 3). Despite this notable difference, common statistical procedures based on dissimilarity indices failed to distinguish between these OT tables (Tutorial 3). Further, correlation coefficients between the raw tables or between distance matrices, calculated using differential weighting of double absences, led to the same conclusion: there were highly significant and strong correlations between the results obtained with the two approaches. OP generated more singleton OTs than MED, which may be observed when an asymmetric (Bray-Curtis) vs. symmetric (Euclidean) dissimilarity coefficient is used (Figures 5A,B, respectively). If OP is to be used to speed up the computation in lieu of MED, the best strategy would be to always use a filtering of the raw tables to avoid the increased generation of singleton OTs by OP.

**Table 2:** **Sample-by-OT tables produced by MED and OP after applying the BSM procedure**.

	**MED**			**OP**		
	**-**	**UC-**	**UU**	**GU-GC**	**GAU-C**	**AUG-U**
HGB_0010	0	2	0	0	2	3
HGB_0011	7	4	1	7	5	0
HGB_0012	25	15	2	25	19	18
HGB_0013	22	24	13	22	41	11
HGB_0014	43	30	25	42	66	9
HGB_0015	18	24	18	18	45	7
HGB_0016	28	35	15	28	54	30
HGB_0017	41	29	6	41	43	16
HGB_0018	26	29	21	24	61	16
HGB_0019	35	25	16	34	49	15
HGB_0023	21	10	6	20	24	11
HGB_0024	52	52	24	51	95	22
HGB_0025	37	34	14	36	58	30

**Table 3:** **Summary of the comparison between 1) OP vs. MED and 2) using the raw compositional table or a compositional table filtered by applying the BSM procedure**.

Type of data	Rawa bundance		BSM	
Method	OP	MED	OP	MED
Table name in the tutorials	TOP0	TM0	TOP_BSM	TM_BSM
Total number of OTs	17	21	3	3
Number of singleton OTs (%)	8 (47%)	3 (14%)	0 (0%)	0 (0%)
Total variance	974.2	543.0	953.3 (97.9%)^$^	472.1 (86.9%)^$^
RV Coefficient	rv: 0.9848^*^		rv: 0.9824^*^	
Mantel test: Bray-Curtis, Euclidean index	r: 0.994^*^, r: 0.981^*^		r: 0.987^*^, r: 0.975^*^	
Correlation of CA ordination plots (Procrustes rotation)	r: 0.787^*^		r: 0.879^*^	
Number of OTs highly correlated (>0.8) to OTs produced with the other approach (% of the total number of OTs) (see Table 1)	11 (64.7%)	12 (57.1%)	2 (66.7%)	3 (100%)

* *P < 0.01*.

$ *percentage referring to the variance in the corresponding raw abundance table*.

One key parameter for ecological comparison and interpretation is the direct correlation among compositional tables (e.g., Gobet et al., 2010), as this ultimately determines the amount of change in community composition. Neither the RV coefficient nor the Mantel test was sensitive enough to capture the fine differences in the highly comparable compositional tables produced by each method (Table 3). However, Procrustes correlation analysis of the correspondence analysis (CA) results was found to be the most sensitive approach (Tutorial 3).

### Comparison of OP and MED for several OTU datasets

A set of 269 OTU FASTA alignments coming from the same study as HGB_0013_GXJPMPL01A3OQX.fasta was submitted to both MED and OP to systematically compare their output (Tutorial 4). MED took about 10 times longer to complete than OP on the same data (about 10 min and less than a min, respectively, on a desktop computer [3.40 GHz, 8 GB RAM, 64-bit Windows 7 OS], when the plotting option was disabled). A total of 217 datasets had Shannon entropy >0.6. The RV coefficients comparing the correlation between the raw OT tables generated by the two approaches ranged from 0.78 to 1.0 (mean of 0.97) and were highly significant. Using CA as a finer approach to detect subtle changes in community composition (see above), 198 OT tables could be represented by a 2D solution and 19 OT tables produced a one-dimensional solution. The former were then used to compare ordination of the samples under the two approaches and 76% of them were found to display significantly related ordination plots, with Procrustes correlation coefficients ranging from 0.54 to 1.0 (mean 0.86).

**Figure 6:**
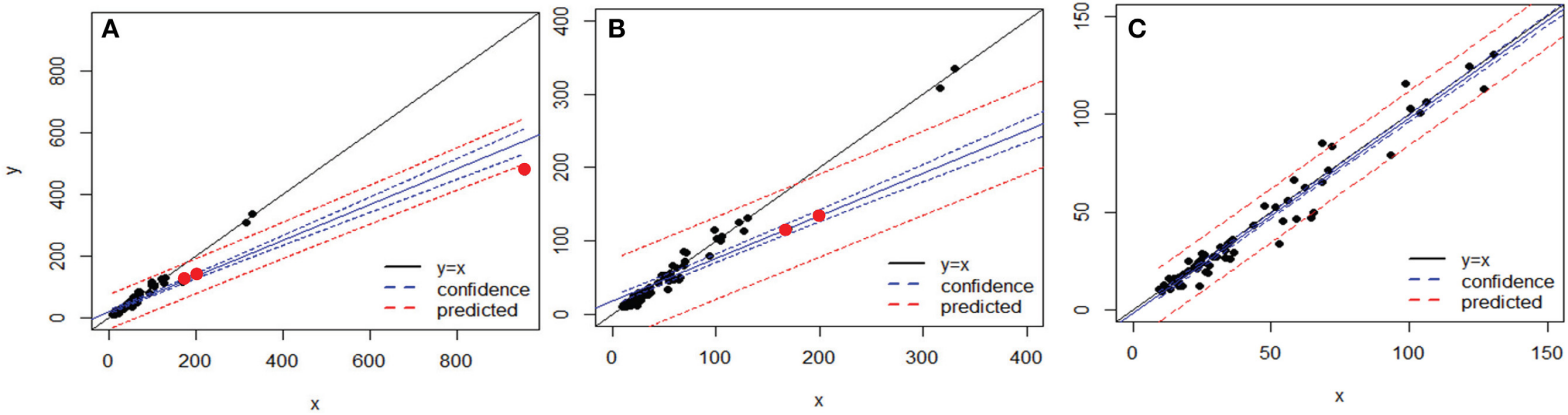
**Comparison of variances obtained by MED (y axis) vs. OP (x axis) across 67 sequence alignments (OTUs), both after BSM filtering. (A)** whole dataset, **(B)** after rescaling to variances below 400 on each axis, and **(C)** after removing the three points that made the y = x line deviate (as red dots in A and B). The blue and red lines represent linear confidence (95%) and prediction lines, respectively.

After applying BSM filtering to MED- and OP-generated tables, only 79 and 123 datasets still contained OTs, respectively, with 67 datasets in common to both techniques. The comparison of the variance in each dataset across the 67 sequence alignments identified three datasets which were mainly responsible for the departure from an exact match between the variances obtained by the two methods for each dataset analyzed (Figure 6). Removing those three datasets, in which OP identified generally higher variance than MED (Tutorial 4), led to a near 1:1 correspondence between the variance obtained by MED and by OP (Figure 6C).

**Figure 7:**
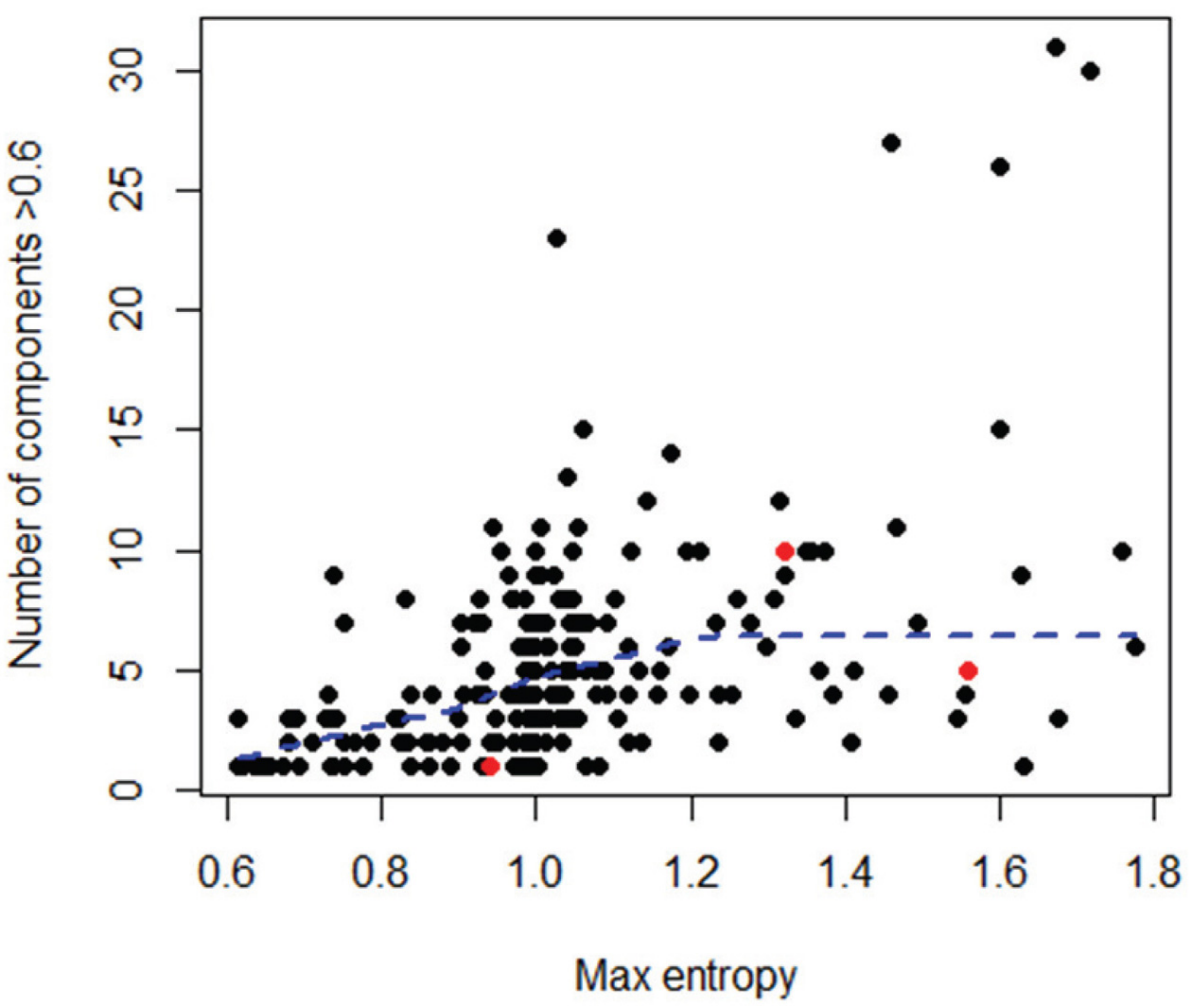
**Comparison of number of components (i.e., high entropy positions) and maximum entropy level for all datasets, considering only the initial sequences in each dataset**. In red are the 3 outlier datasets shown in Figure 6. A LOWESS local fitting line (dotted line) was used to describe the complex shape of the relationship.

To better explore the nature of this discrepancy, the three outlier datasets were further compared to the rest of the datasets in terms of maximum entropy level and number of components in the initial sequence alignments; however, these three datasets did not show any particularly extreme behavior (Figure 7). Likewise, no obvious relationship could be found between the variance in an OT table and either the maximum entropy or number of components found in the initial sequence alignments (Tutorial 4).

The RV coefficients, ranging from 0.79 to 1.0, were very similar to those reported for the methodological comparison based on the raw data. When CA was applied, 25 (48%) out of 57 remaining datasets had a valid 2D representation, from which 19 (i.e., 76%) were significantly correlated across methods with Procrustes coefficients ranging from 0.57 to 1.0 (mean of 0.77). Only seven out of 25 had a Procrustes correlation coefficient > 0.8 (Tutorial 4), thus indicating that few datasets had strong agreement between the CA solutions produced by MED and those produced by OP.

## Conclusions

The initial choice of file “HGB_0013_GXJPMPL01A3OQX.fasta,” which was randomly done, was to some extent unfortunate because that dataset belongs to one of the outlier datasets identified above. When all datasets were used to allow for a more robust methodological comparison, OP seemed to offer a good approach to first screen a large number of sequence datasets (i.e., OTUs), which may then be submitted to MED for more in-depth, and more computationally-demanding, analysis of the existing microdiversity. As demonstrated here, however, the OT tables produced by OP and MED might sometimes not necessarily capture the same ecological information, and this was particularly notable when investigating the fine correspondences between OT abundance and sample mapping in ordination space.

### Conflict of interest statement

The authors declare that the research was conducted in the absence of any commercial or financial relationships that could be construed as a potential conflict of interest.
