# Supplementary material for: The R package otu2ot for implementing the entropy decomposition of nucleotide variation in sequence data
Source: Front Microbiol. 2014 Nov 14;5:601. doi: 10.3389/fmicb.2014.00601 (PMC4231947; doi:10.3389/fmicb.2014.00601)
Supplement: Supplementary file 1 [file Presentation1.ZIP › Supplementary Material/Tutorial 0 - Installing the otu2ot package.pdf]

Tutorial 0: Installing the package from the provided zip or tar.gz files

In a R console

```
setwd(“Directory/where/the/packageFiles/are/found”)
install.packages("otu2ot_1.4.tar.gz", repos = NULL, type = "source")
```

or using the package.zip version

```
install.packages("otu2ot_1.4.zip", repos = NULL)
```

```
package ‘otu2ot’ successfully unpacked and MD5 sums checked
```

#load the package

```
library(otu2ot)
```

#get some help on the functions and datasets.

```
help(package = otu2ot)
```
