# Supplementary material for: The R package otu2ot for implementing the entropy decomposition of nucleotide variation in sequence data
Source: Front Microbiol. 2014 Nov 14;5:601. doi: 10.3389/fmicb.2014.00601 (PMC4231947; doi:10.3389/fmicb.2014.00601)

Tutorial 1: Applying MED to sequences from one OTU

```
library(otu2ot)
#help(package = otu2ot)
```

1) option a) The file is found in the working directory as a FASTA file containing aligned sequences.

```
File="HGB_0013_GXJPMPL01A3OQX.fasta"
```

```
OT.seq.concat1 <- MED(File,minseq=21,entropymin=0.6,Plot=TRUE)
```

```
#Position: 242
#            - A C      G     U
#Nber 355.00 4 4 247.00 565.0
#Prop   0.31 0 0   0.22   0.5
```

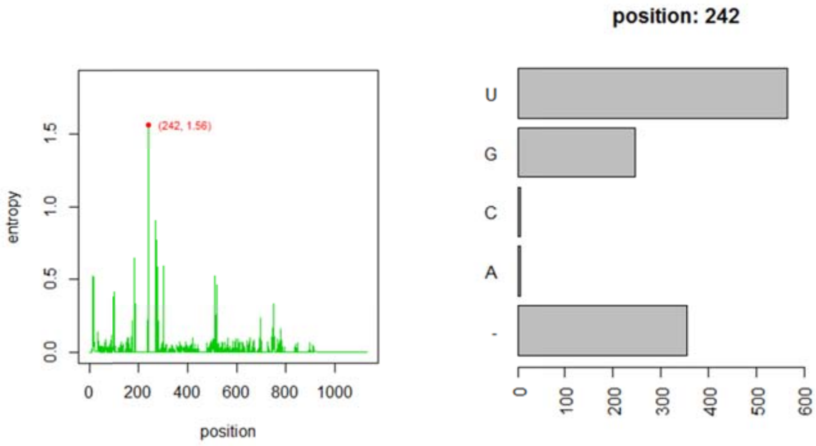

The plots are only for the first entropy profile when all sequences are considered.

```
head(OT.seq.concat1)
```

```
      1        2        3        4        5        6
  "UC-"    "UC-" "GCAAAU" "GCA-AU" "GCAAAU"      "-"
```

```
system.time( MED(File,minseq=21,entropymin=0.6,Plot=FALSE))
```

#user system elapsed

#10.81   0.03   10.92

Option b) Importing first the sequences, and then applying MED, via the MEDMat function

```
Aln.list<- ImportFastaAlignment(File)
Names <-  Aln.list[[1]]
Sequences <- toupper(Aln.list[[2]])
OT.seq.concat <- MEDMat(AlignedSequences=Sequences,minseq=21,
entropymin=0.6, Plot=TRUE)
```

2) Retrieve the sample information from the FASTA headers directly. To see if the length of the substring in the FASTA headers is correct, use:

```
GetEnvironmentDatafromFileR(File,Start=2,Stop=9,test=TRUE)
```

```
[1] "HGB_0010"
```

Then apply it to all headers:

```
ENV <-
GetEnvironmentDatafromFileR(File,Start=2,Stop=9,test=FALSE)
```

3) build the Sample-by-OT table and filter by abundance too if needed.

```
Table0 <- SampleXOT_Table(
     OT.seq.concat=OT.seq.conca
     t,
     ENV=ENV,
     mosaicPlot=TRUE,
     filterByMinAbund= 0
)
```

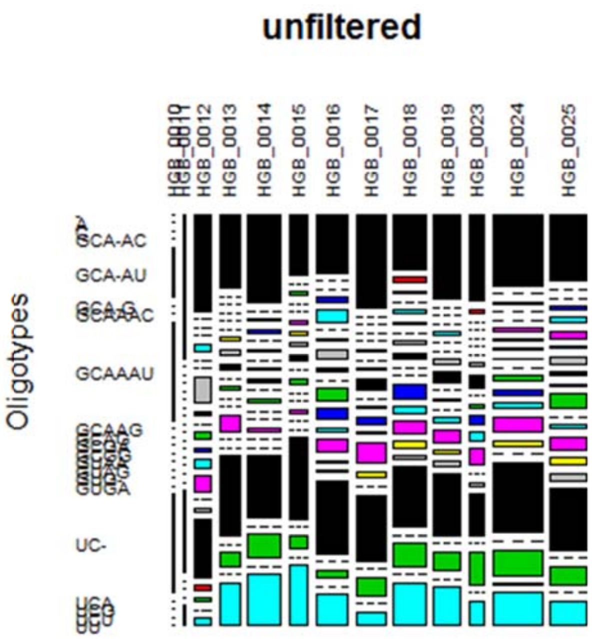


```
Table10 <- SampleXOT_Table(
      OT.seq.concat=OT.seq.concat,
     ENV=ENV,
     mosaicPlot=TRUE,
     filterByMinAbund= 10
)
```

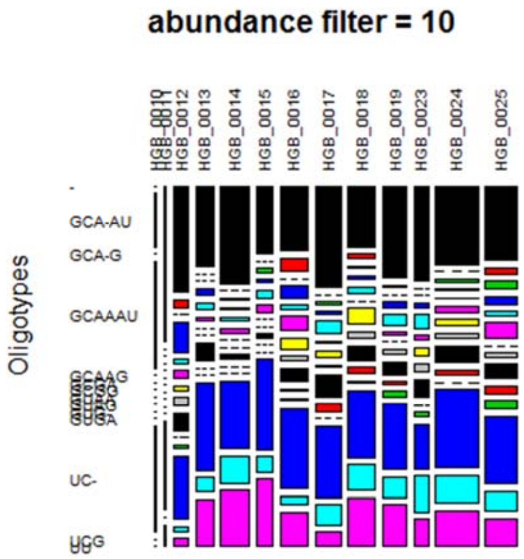


```
str(Table0)
```

```
List of 1
 $ SamplexOT.table: int [1:13, 1:21] 0 7 25 22 43 18 28 41 26 35 ...
  ..- attr(*, "dimnames")=List of 2
  .. ..$ Samples   : chr [1:13] "HGB_0010" "HGB_0011" "HGB_0012" "HGB_0013" ..
.
  .. ..$ Oligotypes: chr [1:21] "-" "A" "C" "GCA-AC" ...
```

Which OT in the raw table have abundances higher than predicted by using the Broken-Stick model approach?

```
OTAbund <- colSums(Table0[[1]])
#overall abundance for each OT

OTAbund_BSM <- Count.BrokenStick(OTAbund, Plot = TRUE)
OTAbund_BSM
$Table
                   -       UC-        UU       UCG      GUAG    GCAAAU      GCGA
Observed  30.21277 26.63830 13.70213 7.574468 5.106383 2.468085 2.382979
FromModel 17.35885 12.59695 10.21599 8.628692 7.438216 6.485835 5.692184
             GCAAG      GCGG      GUAA      GUG-    GCA-AU     GCA-G      GUGA
Observed  2.297872 1.872340 1.531915 1.361702 1.276596 0.8510638 0.8510638
FromModel 5.011912 4.416674 3.887574 3.411383 2.978483 2.5816573 2.2153569
             GCA-AC    GCAAAC         A         C       GCAG        UCA
Observed  0.5106383 0.4255319 0.3404255 0.3404255 0.08510638 0.08510638
FromModel 1.8752208 1.5577605 1.2601415 0.9800294 0.71547917 0.46485261
                 UCU
Observed  0.08510638
FromModel 0.22675737

$HigherThanBSM
[1] "-"   "UC-" "UU"
```

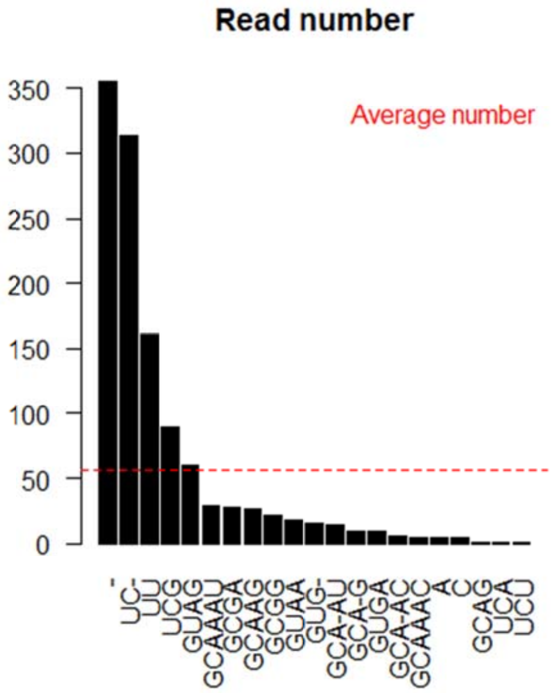

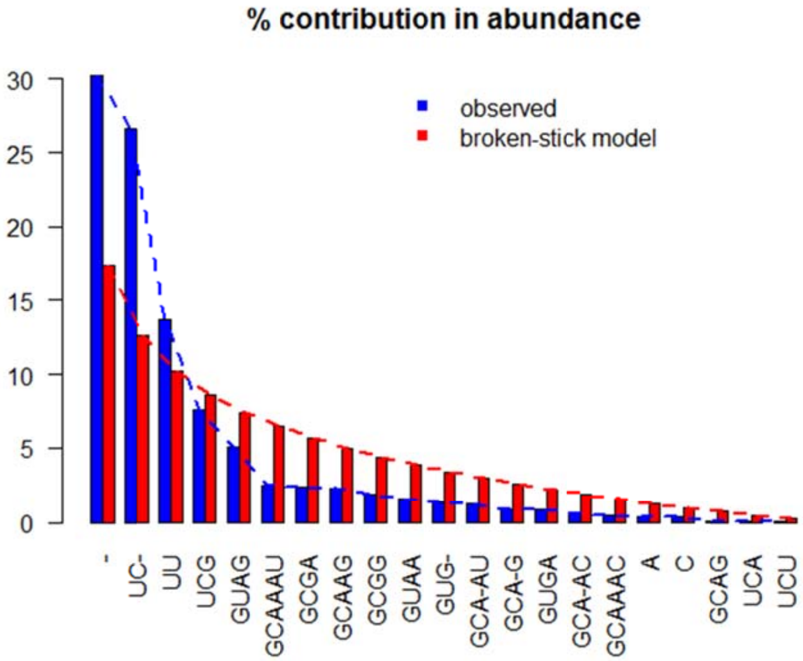

Conclusions: here only three OT have relative abundances higher than predicted by a random partition of sequence abundance.

```
Table0_BSM <- Table0[[1]][,OTAbund_BSM$HigherThanBSM]
Table0_BSM
          Oligotypes
Samples     - UC- UU
  HGB_0010  0   2  0
  HGB_0011  7   4  1
  HGB_0012 25  15  2
  HGB_0013 22  24 13
  HGB_0014 43  30 25
```

```
  HGB_0015 18   24 18
  HGB_0016 28   35 15
  HGB_0017 41   29  6
  HGB_0018 26   29 21
  HGB_0019 35   25 16
  HGB_0023 21   10  6
  HGB_0024 52   52 24
  HGB_0025 37   34 14
```

```
mosaicplot(Table0_BSM,col=1:ncol(Table0_BSM),
            main="BSM",xlab="",las=2,cex=0.6)
```

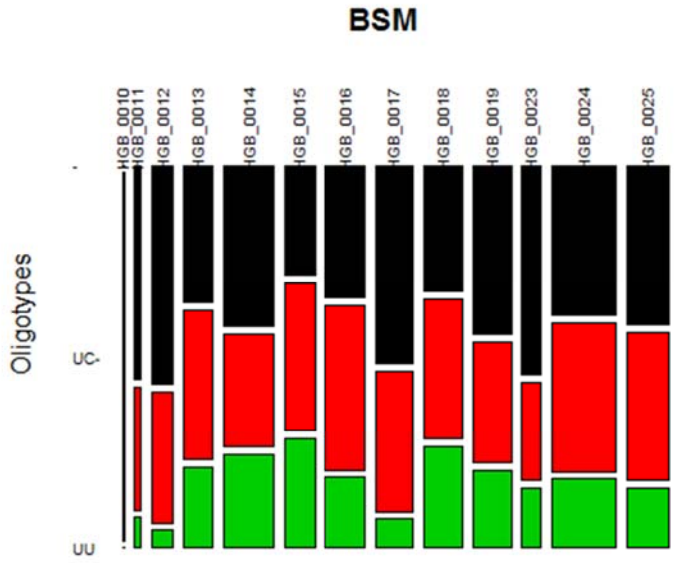

Supplement: Supplementary file 1 [file Presentation1.ZIP › Supplementary Material/Tutorial 1 - MED of one FASTA alignment file.pdf]
