# Supplementary material for: The R package otu2ot for implementing the entropy decomposition of nucleotide variation in sequence data
Source: Front Microbiol. 2014 Nov 14;5:601. doi: 10.3389/fmicb.2014.00601 (PMC4231947; doi:10.3389/fmicb.2014.00601)
Supplement: Supplementary file 1 [file Presentation1.ZIP › Supplementary Material/Tutorial 2 - One-Pass profiling on one FASTA alignment.pdf]

Tutorial 2: Applying One-Pass profiling to sequences from one OTU

```
library(otu2ot)
#help(package = otu2ot)
```

1) option a) The file is found in the working directory as a FASTA file containing aligned sequences.

```
File=" HGB_0013_GXJPMPL01A3OQX.fasta"

OnePass <- OnePassProfiling(File="HGB_0013_GXJPMPL01A3OQX.fasta",
  minseq=21,
  entropymin=0.6,
  Plot=TRUE
)
```

```
Position: 185
          A      G
Nber 193.00 982.00
Prop   0.17   0.87

Position: 241
         A C G      U
Nber 568.0 3 5 599.00
Prop   0.5 0 0   0.53

Position: 242
          - A C      G     U
Nber 355.00 4 4 247.00 565.0
Prop   0.31 0 0   0.22   0.5

Position: 271
          - A      G
Nber 818.00 2 355.00
Prop   0.72 0   0.31

Position: 272
          C G      U
Nber 981.00 1 193.00
Prop   0.87 0   0.17
```

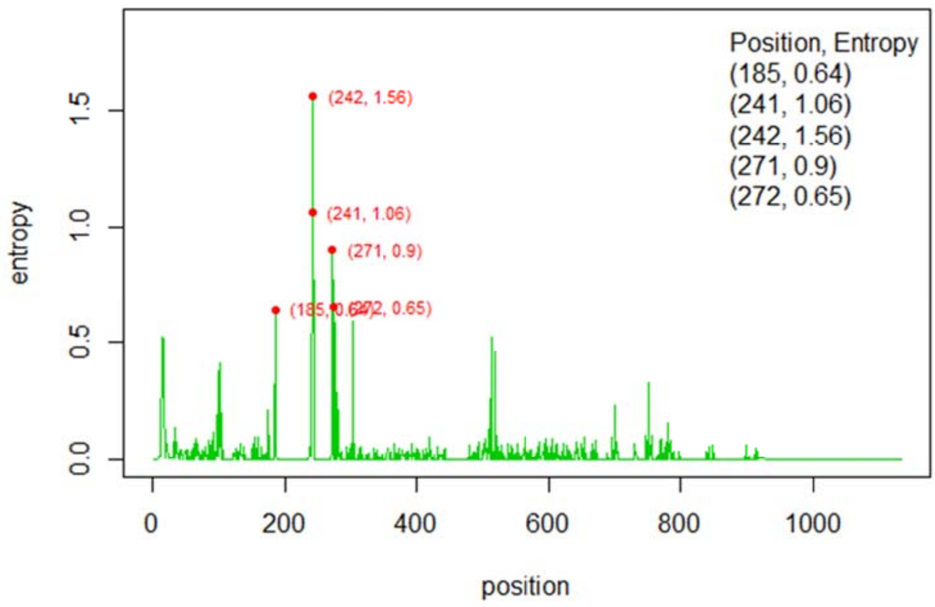

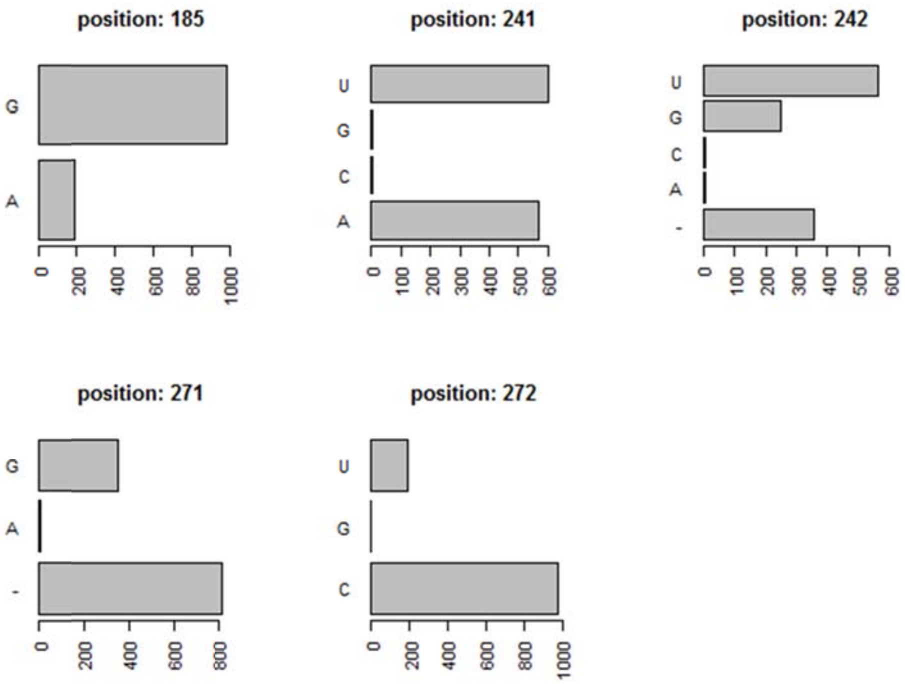

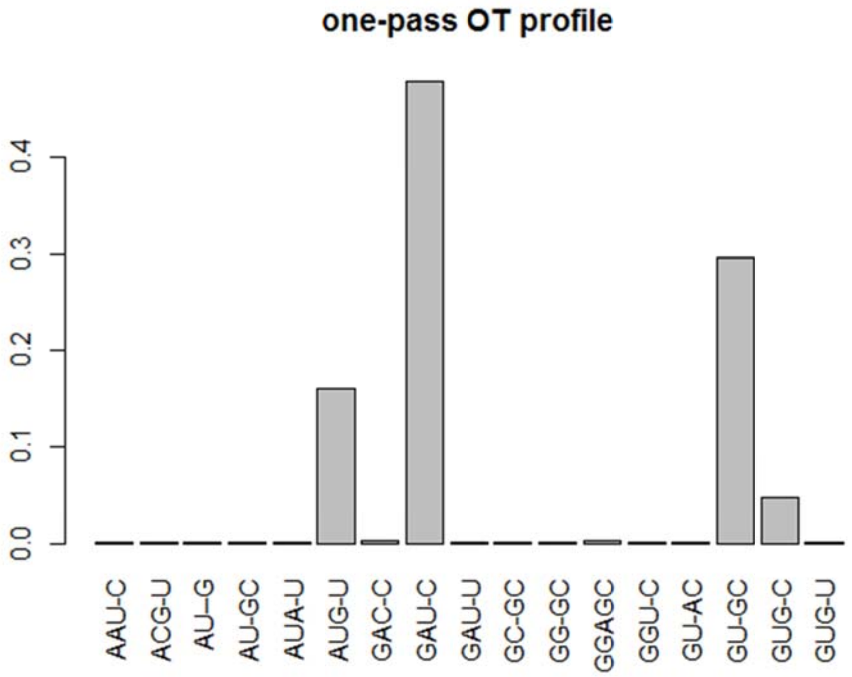


```
str(OnePass)
```

```
List of 3
 $ OT.seq.concat: Named chr [1:1175] "GAU-C" "GAU-C" "AUG-U" "AUG-U" ...
  ..- attr(*, "names")= chr [1:1175] "1" "2" "3" "4" ...
 $ OT.count     : 'table' int [1:17(1d)] 1 1 1 1 1 188 4 562 1 2 ...
  ..- attr(*, "dimnames")=List of 1
  .. ..$ OT.seq.concat: chr [1:17] "AAU-C" "ACG-U" "AU--G" "AU-GC" ...
 $ OT.freq      : table [1:17(1d)] 0.000851 0.000851 0.000851 0.000851 0.00085
1 ...
```

```
  ..- attr(*, "dimnames")=List of 1
  .. ..$ OT.seq.concat: chr [1:17] "AAU-C" "ACG-U" "AU--G" "AU-GC" ...
```

```
system.time(
OnePassProfiling(File="HGB_0013_GXJPMPL01A3OQX.fasta",
  minseq=21,
  entropymin=0.6,
  Plot=TRUE
     ))
user  system elapsed
3.00    0.02    3.01
```

Option b) Importing first the sequences, and then applying the `OnePassProfilingMat` function

```
Aln.list<- ImportFastaAlignment(File)
Names <-  Aln.list[[1]]
Sequences <- toupper(Aln.list[[2]])
OT.seq.concat2 <- OnePassProfilingMat
(AlignedSequences=Sequences,minseq=21,  entropymin=0.6,
Plot=TRUE)
```

#not shown

```
system.time(OnePassProfiling(File="HGB_0013_GXJPMPL01A3OQX.fasta"
,
  minseq=21,
  entropymin=0.6,
  Plot=TRUE
     ))
user  system elapsed
0.32    0.00    0.32
```

2) Retrieve the sample information from the FASTA headers directly. To see if the length of the substring in the FASTA headers if correct, use:

```
GetEnvironmentDatafromFileR(File,Start=2,Stop=9,test=TRUE)
```

```
[1] "HGB_0010"
```

Then apply it to all headers:

```
ENV <-
GetEnvironmentDatafromFileR(File,Start=2,Stop=9,test=FALSE)
```

3) build the Sample-by-OT table and filter by abundance too if needed.

```
Table0 <- SampleXOT_Table(    OT.seq.concat=OnePass[[1]],
     ENV=ENV,
     mosaicPlot=TRUE,
     filterByMinAbund= 0
)
```

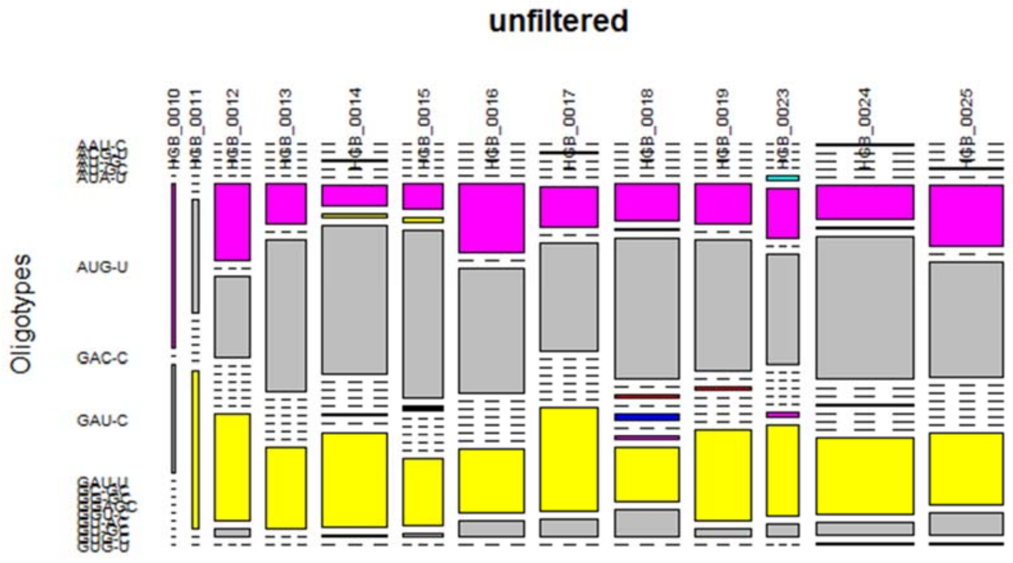


```
str(Table0)
```

```
List of 1
 $ SamplexOT.table: int [1:13, 1:17] 0 0 0 0 0 0 0 0 0 0 ...
  ..- attr(*, "dimnames")=List of 2
  .. ..$ Samples   : chr [1:13] "HGB_0010" "HGB_0011" "HGB_0012" "HGB_0013"...
  .. ..$ Oligotypes: chr [1:17] "AAU-C" "ACG-U" "AU--G" "AU-GC" ...
```

Which OT in the raw table have abundances higher than predicted by using the Broken-Stick model approach?

```
OTAbund <- colSums(Table0[[1]])
#overall abundance for each OT

OTAbund_BSM <- Count.BrokenStick(OTAbund, Plot = TRUE)
```

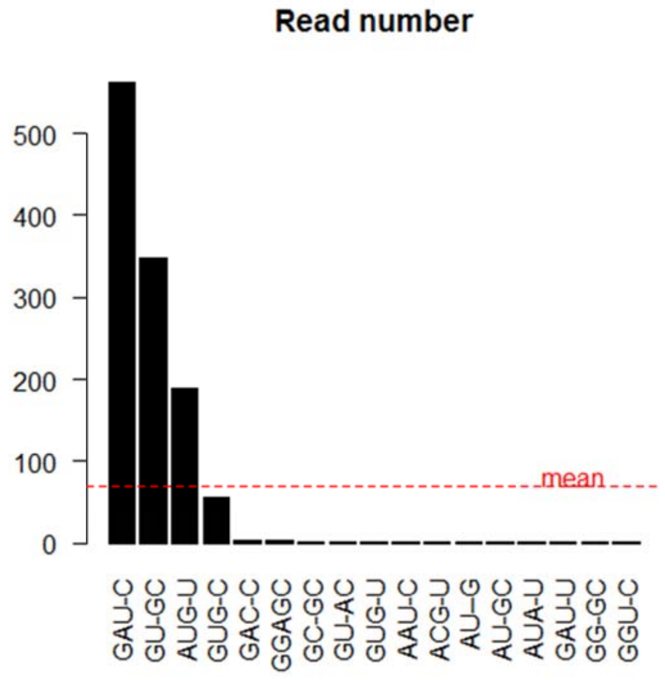

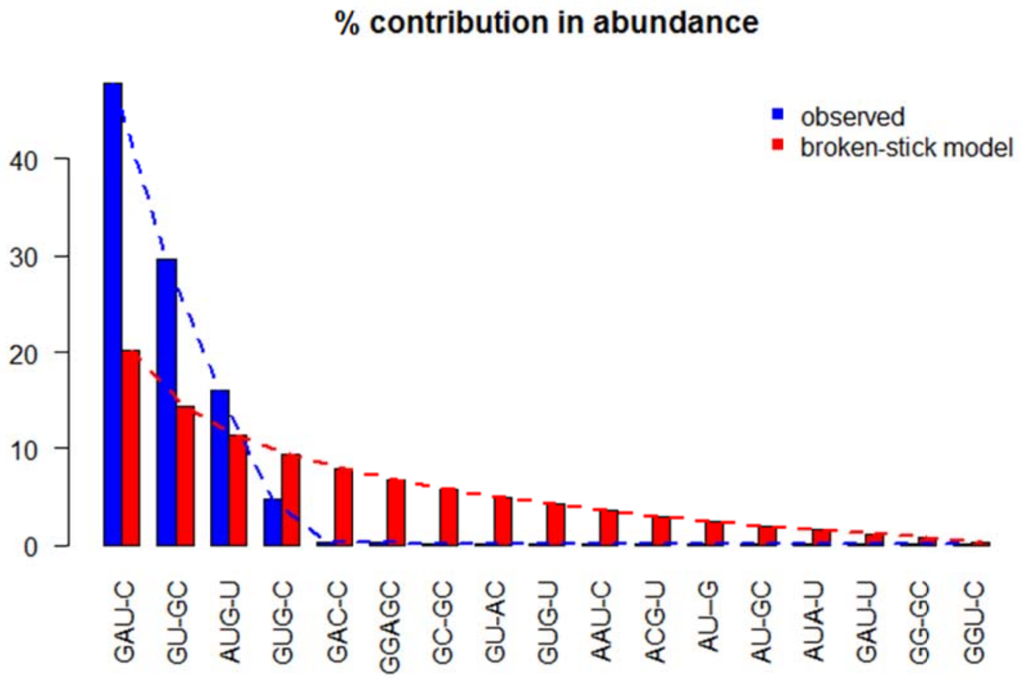

OTAbund_BSM

```
$Table
             GAU-C     GU-GC     AUG-U     GUG-C      GAC-C      GGAGC
Observed  47.82979 29.61702 16.00000 4.765957 0.3404255 0.2553191
FromModel 20.23266 14.35031 11.40913 9.448348 7.9777599 6.8012893
              GC-GC      GU-AC      GUG-U       AAU-C       ACG-U
Observed  0.1702128 0.1702128 0.1702128 0.08510638 0.08510638
FromModel 5.8208972 4.9805611 4.2452669 3.59167217 3.00343687
               AU--G      AU-GC      AUA-U      GAU-U      GG-GC
Observed  0.08510638 0.08510638 0.08510638 0.08510638 0.08510638
FromModel 2.46867752 1.97848144 1.52599275 1.10582468 0.71366782
               GGU-C
Observed  0.08510638
FromModel 0.34602076

$HigherThanBSM
[1] "GAU-C" "GU-GC" "AUG-U"
```

Conclusions: here only three OT have relative abundances higher than predicted by a random partition of sequence abundance.

Table0_BSM <- Table0[[1]][,OTAbund_BSM$HigherThanBSM]
Table0_BSM

```
          Oligotypes
Samples    GAU-C GU-GC AUG-U
  HGB_0010     2     0     3
  HGB_0011     5     7     0
  HGB_0012    19    25    18
  HGB_0013    41    22    11
  HGB_0014    66    42     9
  HGB_0015    45    18     7
  HGB_0016    54    28    30
  HGB_0017    43    41    16
  HGB_0018    61    24    16
  HGB_0019    49    34    15
  HGB_0023    24    20    11
  HGB_0024    95    51    22
```

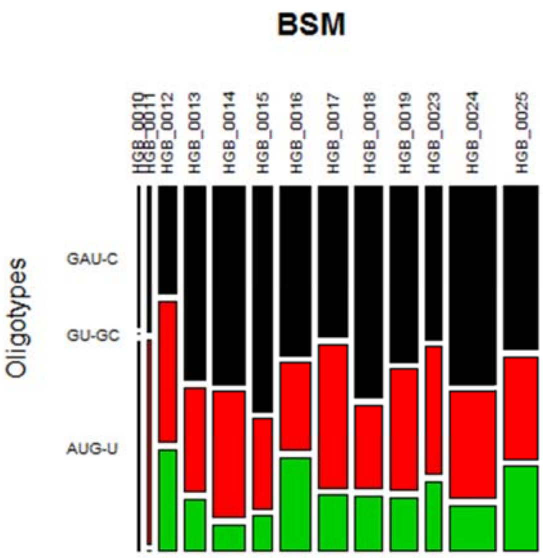

```
  HGB_0025    58    36    30
```

```
mosaicplot(Table0_BSM,col=1:ncol(Table0_BSM),
        main="BSM",xlab="",las=2,cex=0.6)
```
