# Supplementary material for: The R package otu2ot for implementing the entropy decomposition of nucleotide variation in sequence data
Source: Front Microbiol. 2014 Nov 14;5:601. doi: 10.3389/fmicb.2014.00601 (PMC4231947; doi:10.3389/fmicb.2014.00601)
Supplement: Supplementary file 1 [file Presentation1.ZIP › Supplementary Material/Tutorial 3 - One-Pass profiling vs. MED on one FASTA alignment.pdf]

Tutorial 3: One-Pass profiling (OP) vs. MED applied to one FASTA alignment (one OTU)

```
library(otu2ot)
help(package = otu2ot)
```

1) option a) The file is found in the working directory as a FASTA file containing aligned sequences.

```
File=" HGB_0013_GXJPMPL01A3OQX.fasta" #1175 sequences, 1133 positions
```

OnePassResults <- `OnePassProfiling(File="HGB_0013_GXJPMPL01A3OQX.fasta",` minseq=21, entropymin=0.6,Plot=TRUE)

```
MEDResults <- MED(File,minseq=21,entropymin=0.6,Plot=TRUE)
```

#timing

```
system.time(OnePassProfiling(File, minseq=21,entropymin=0.6, Plot=TRUE))
user  system elapsed
3.10    0.01    3.12
```

```
system.time(MED(File,minseq=21,entropymin=0.6,Plot=TRUE))
user  system elapsed
12.18    0.03   12.25
```

get the ENV information (i.e. sample origin) from the FASTA header

```
ENV <- GetEnvironmentDatafromFileR(File,Start=2,Stop=9,test=FALSE)
table(ENV)
ENV
HGB_0010 HGB_0011 HGB_0012 HGB_0013 HGB_0014 HGB_0015 HGB_0016
       5       12       64       74      121       73      119
HGB_0017 HGB_0018 HGB_0019 HGB_0023 HGB_0024 HGB_0025
     108      119      102       60      181      137
```

#########################
build the Sample by OT table.

```
Table.MED.0 <- SampleXOT_Table(
 OT.seq.concat=MEDResults,
  ENV=ENV,
  mosaicPlot=TRUE,
  filterByMinAbund= 0
)
```

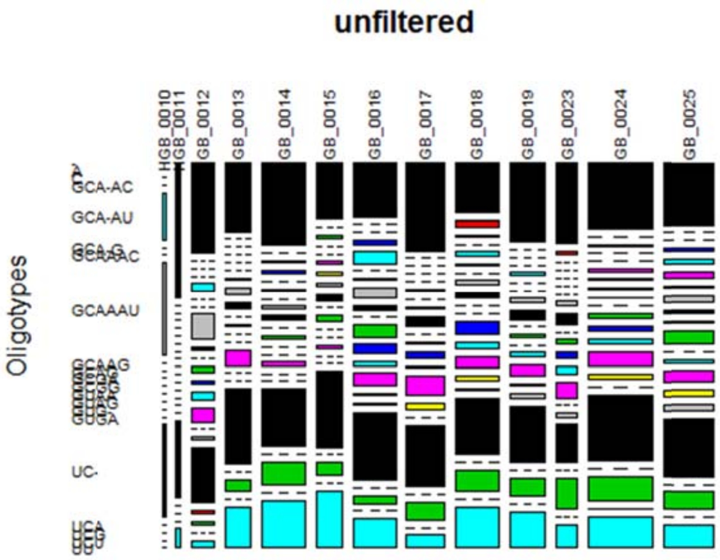

```
Table.OnePass.0 <- SampleXOT_Table(
  OT.seq.concat=OnePassResults[[1]],
  ENV=ENV,
  mosaicPlot=TRUE,
  filterByMinAbund= 0
)
```

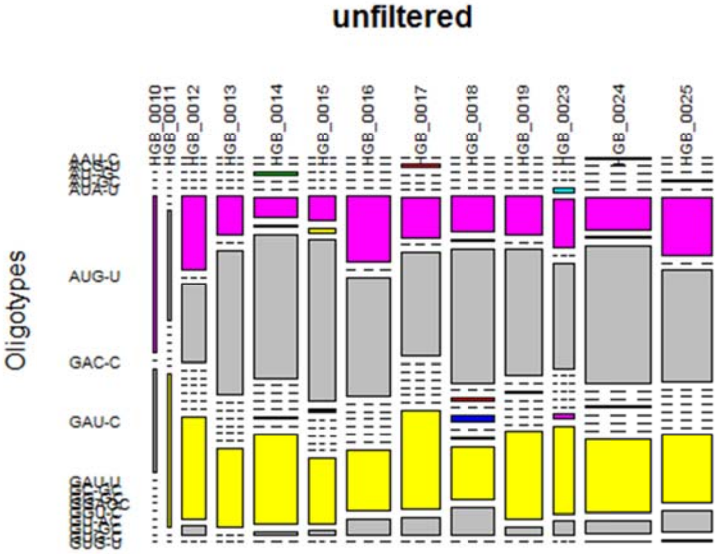

Comparison of the two methods based on unfiltered tables

```
TM0 <- Table.MED.0[[1]]
TM0[,names(sort(colSums(TM0),decreasing = TRUE))]
```
#sorting by decreasing OT abundance

Samples	-	UC-	UU	UCG	GUAG	GCAAAU	GCGA	GCAAG	GCGG	GUAA	GUG-	GCA-AU	GCA-G	GUGA	GCA-AC	GCAAAC	A	C	GCAG	UCA	UCU
HGB_0010	0	2	0	0	0	2	0	0	0	0	0	1	0	0	0	0	0	0	0	0	0
HGB_0011	7	4	1	0	0	0	0	0	0	0	0	0	0	0	0	0	0	0	0	0	0
HGB_0012	25	15	2	1	4	7	2	1	1	2	0	2	0	1	0	0	0	0	0	1	0
HGB_0013	22	24	13	4	5	2	1	2	0	0	0	0	0	0	0	1	0	0	0	0	0
HGB_0014	43	30	25	12	2	1	2	2	0	0	0	0	1	0	2	0	0	1	0	0	0
HGB_0015	18	24	18	4	1	1	2	2	0	0	0	0	1	0	0	1	0	1	0	0	0
HGB_0016	28	35	15	4	6	5	6	2	5	2	1	6	0	1	2	1	0	0	0	0	0
HGB_0017	41	29	6	8	9	1	1	5	3	1	3	0	1	0	0	0	0	0	0	0	0
HGB_0018	26	29	21	11	6	2	0	3	7	3	3	2	1	1	0	0	3	1	0	0	0
HGB_0019	35	25	16	8	5	2	1	4	0	2	1	1	0	2	0	0	0	0	0	0	0
HGB_0023	21	10	6	8	4	1	1	3	2	2	0	0	0	1	0	0	1	0	0	0	0
HGB_0024	52	52	24	19	11	1	4	1	4	4	4	0	2	0	0	1	0	1	0	0	1
HGB_0025	37	34	14	10	7	4	8	2	0	2	4	3	4	4	2	1	0	0	1	0	0

```
TOP0 <- Table.OnePass.0[[1]]
TOP0[,names(sort(colSums(TOP0),decreasing = TRUE))]
```

Samples	GAU-C	GU-GC	AUG-U	GUG-C	GAC-C	GGAGC	GC-GC	GU-AC	GUG-U	AAU-C	ACG-U	AU--G	AU-GC	AUA-U	GAU-U	GG-GC	GGU-C
HGB_0010	2	0	3	0	0	0	0	0	0	0	0	0	0	0	0	0	0
HGB_0011	5	7	0	0	0	0	0	0	0	0	0	0	0	0	0	0	0
HGB_0012	19	25	18	2	0	0	0	0	0	0	0	0	0	0	0	0	0
HGB_0013	41	22	11	0	0	0	0	0	0	0	0	0	0	0	0	0	0
HGB_0014	66	42	9	1	1	0	0	0	0	0	0	1	0	0	0	0	1
HGB_0015	45	18	7	1	1	0	0	0	0	0	0	0	0	0	1	0	0
HGB_0016	54	28	30	7	0	0	0	0	0	0	0	0	0	0	0	0	0
HGB_0017	43	41	16	7	0	0	0	0	0	0	1	0	0	0	0	0	0
HGB_0018	61	24	16	12	1	3	1	1	0	0	0	0	0	0	0	0	0
HGB_0019	49	34	15	3	0	0	1	0	0	0	0	0	0	0	0	0	0
HGB_0023	24	20	11	3	0	0	0	1	0	0	0	0	0	1	0	0	0
HGB_0024	95	51	22	9	1	0	0	0	1	1	0	0	0	0	0	1	0
HGB_0025	58	36	30	11	0	0	0	0	1	0	0	0	1	0	0	0	0

```
dim(TM0)
[1] 13 21

dim(TOP0)
[1] 13 17
```

Comparing the abundance of each OT provided by the two techniques:

```
colSums(TM0[,names(sort(colSums(TM0),decreasing = TRUE))])
-     UC-      UU     UCG    GUAG GCAAAU    GCGA   GCAAG    GCGG
   355     313     161      89      60      29      28      27      22
  GUAA    GUG-  GCA-AU   GCA-G    GUGA  GCA-AC  GCAAAC       A       C
    18      16      15      10      10       6       5       4       4
  GCAG     UCA     UCU
     1       1       1
colSums(TOP0[,names(sort(colSums(TOP0),decreasing = TRUE))])
GAU-C GU-GC AUG-U GUG-C GAC-C GGAGC GC-GC GU-AC GUG-U AAU-C
  562   348   188    56     4     3     2     2     2     1
ACG-U AU--G AU-GC AUA-U GAU-U GG-GC GGU-C
    1     1     1     1     1     1     1
```

So it appears that OP table has fewer number of columns (i.e. OT), as expected, but also displays more singleton OT. The two tables have the same total number of sequences of course:

```
sum(TM0)
[1] 1175
sum(TOP0)
[1] 1175
```

Total variance for each dataset:

```
sum(apply(TM0,2,var))
[1] 542.9615
sum(apply(TOP0,2,var))
[1] 974.1795
```

So the MED and the OP approaches do no yield the same total variance among the OT. Noticeably, the OP approach leads to higher community variance.

Yet, the direct correlation of the two compositional tables using the RV coefficient is high and significant. The RV coefficient (Escouffier 1973) is a measure of relationship between two sets of variables and it is based on the principle that two sets of variables are perfectly correlated if there exists an orthogonal transformation that makes the two sets coincide.

```
require(FactoMineR)
coeffRV(X=TM0,Y=TOP0)[c(1,6)] #rv and P value
$rv
[1] 0.984826
$p.value
[1] 6.545674e-05
```

The dissimilarity values among samples based on the two tables are also very similar: Using a asymmetric dissimilarity coefficient (e.g Bray-Curtis) that gives no importance to double “0” in the data when computing sample (dis)similarity or using a symmetric coefficient (e.g. Euclidean) that gives as much importance to double absences than any other values (Legendre and Legendre 1998), one can find:

```
require(vegan)
mantel(vegdist(TM0), vegdist(TOP0))#Bray-Curtis
Mantel statistic r: 0.994
      Significance: 0.001
plot.lm.ci1(x=as.numeric(vegdist(TM0)),y=as.numeric(vegdist(TOP0)),main="v
egdist (TM0~TOP0)")


mantel(dist(TM0), dist(TOP0))# Euclidean
Mantel statistic r: 0.9814
      Significance: 0.001
```

```
plot.lm.ci1(x=as.numeric(dist(TM0)),y=as.numeric(dist(TOP0)),main="dist(TM
0~TOP0)")
```

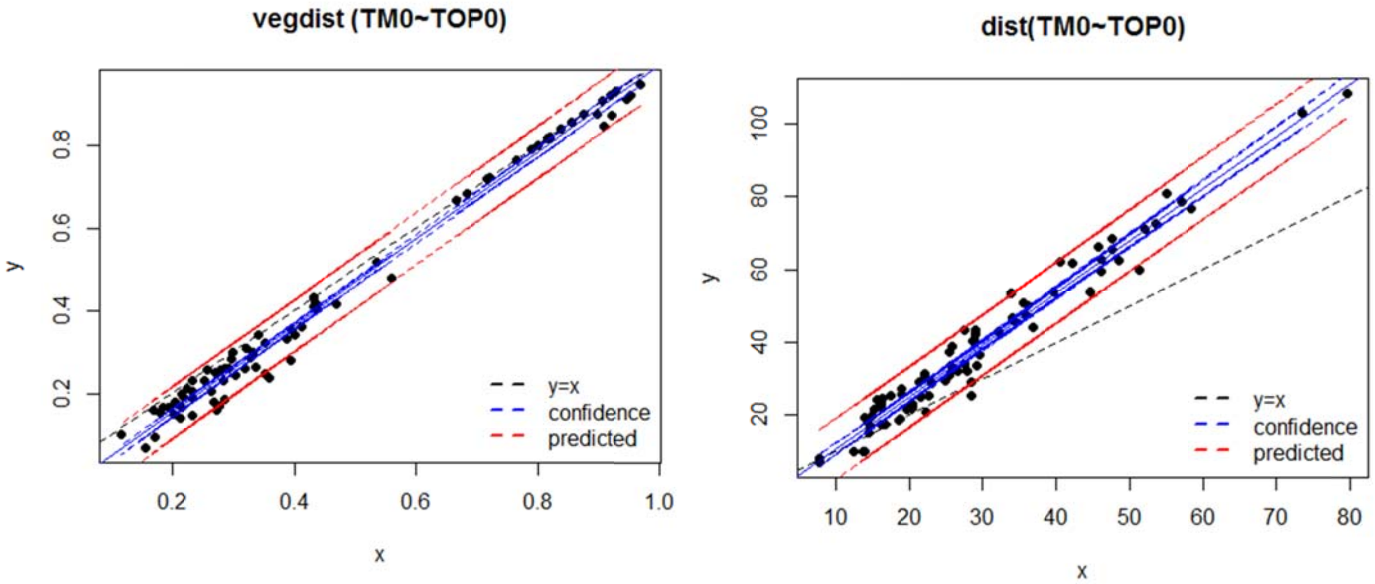

The changes in community structure in the composition tables produced by MED or by OP are very similar to each other, especially if one does not give weight to double absences, as generally done when dealing with compositional data, where double "species' absence (i.e. absence at two sites of a species) should not be taken as indicator for site ecological similarity (e.g. Legendre and Legendre 1998). There is overall more dissimilarities between samples when MED is used as compared to OP, especially when using symmetric coefficients (here Euclidean) that give importance to double absences.

Comparison of the ordination plots of the two tables

```
NMDS.TM0= metaMDS(TM0)
Stress:     8.92583e-05
```

trivial solution, as it seems that the data is very (too) simple to be embedded in 2-dimensional space.

```
NMDS.TOP0= metaMDS(TOP0)
Stress:     0.09993686
```

```
plot(NMDS.TOP0,display="sites",xaxt="n",yaxt="n",main="OP")
text(NMDS.TOP0$points[,1:2],rownames(TOP0),cex=0.5,pos=4)
plot(NMDS.TM0,display="sites",xaxt="n",yaxt="n",main="MED")
text(NMDS.TM0$points[,1:2],rownames(TM0),cex=0.5,pos=4)
```

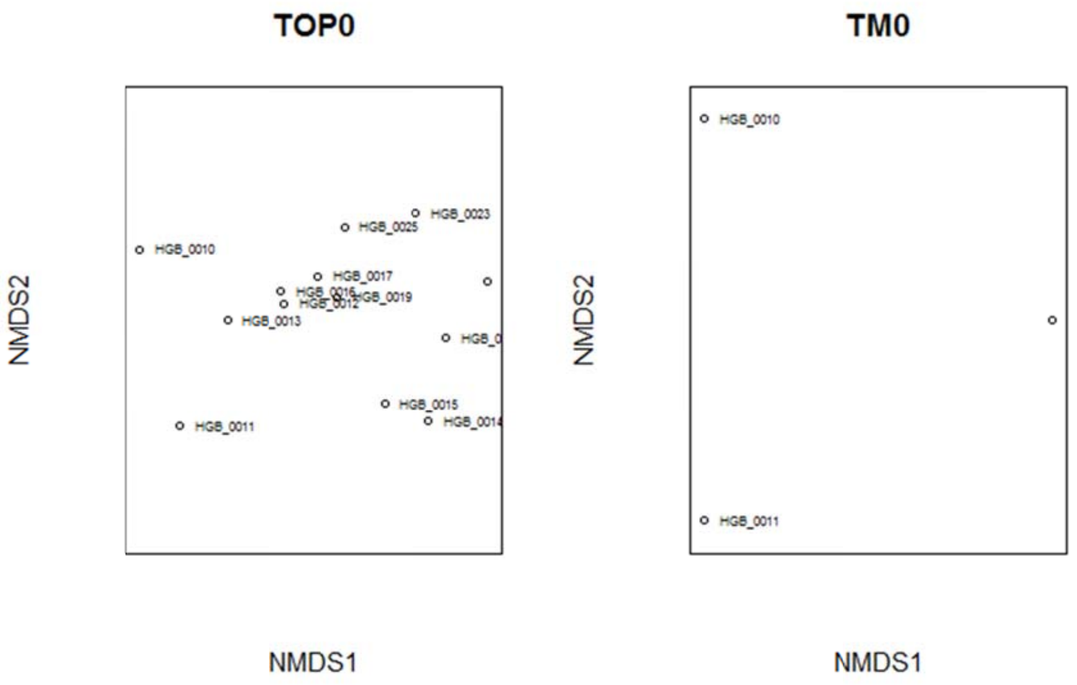

Function *procrustes* rotates a configuration to maximum similarity with another configuration. Function *protest* tests the non-randomness (‘significance’) between two configurations.

```
P.NMDS.0 <- protest(NMDS.TOP0,NMDS.TM0)
Correlation in a symmetric Procrustes rotation: 0.666
Significance:  0.001
Based on 999 permutations.
```

```
plot(P.NMDS.0)
text(P.NMDS.0, display = c("rotated"),cex=0.5,col="blue") # in blue TM0
text(P.NMDS.0, display = c("target"),cex=0.5,col="red")  # in red TOP0
```

The superimposition of the two ordination plots reveals that indeed the overall patterns of sample dissimilarities are kept, but the TM0 dataset could not resolve further the differences between all samples from HGB_0012 to JGB_0025, the latter all plotted on the same coordinates. This may be due to the fact that MED led to a reduced amount of variance in the original sample by OT table, as compared to OP.

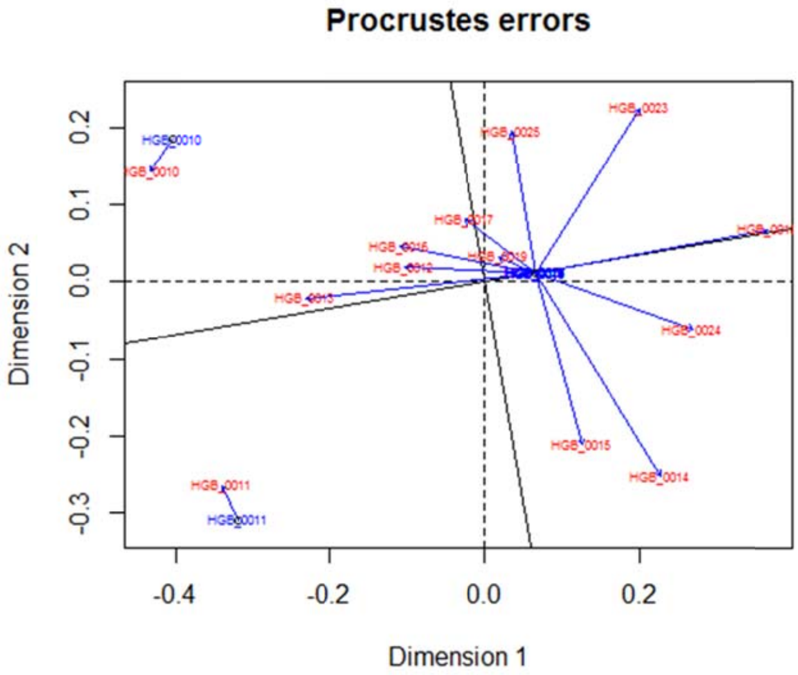

Maybe NMDS was not the best technique to analyse the MED data, especially indicated by the extremely low stress value. Let’s try correspondence analysis (CA) to better resolve the finer correspondence between OT abundance and sample:

```
CA.TMO <- cca(TM0)
  plot(CA.TMO,main="CA.TM0",xlim=c(-2,9),ylim=c(-4,2))
CA.TOPO <- cca(TOP0)
  plot(CA.TOPO,main="CA.TOP0",xlim=c(-3,4))
```

What is interesting too with a CA representation is that the position of one OT indicates its relative abundance in samples based on the proximity of the OT position and the sample position in the CA plot (hence the name “correspondence analysis”).

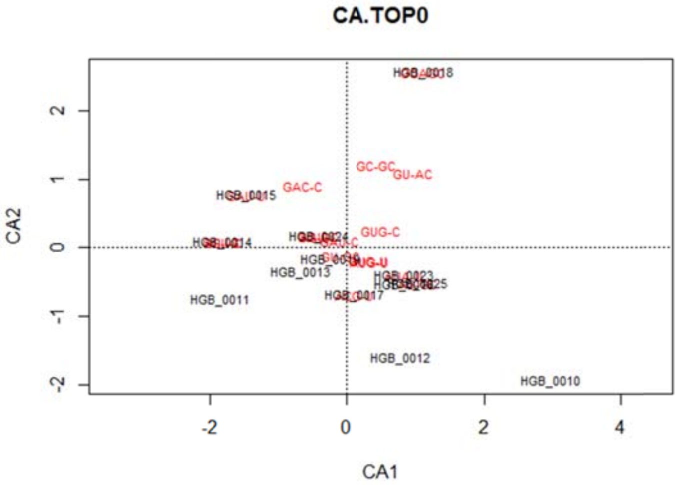

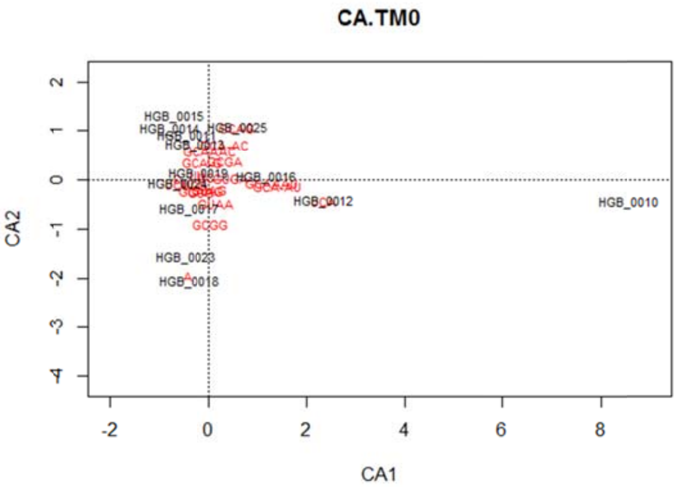

Here it is clear that the MED data (TM0) are better visualized with CA instead than with NMDS.

A Procrutes analysis to correlate the two ordination results gives:

```
P.CA.0 <- protest(CA.TOPO,CA.TMO)
Correlation in a symmetric Procrustes rotation: 0.7868
Significance:  0.001
Based on 999 permutations.

plot(P.CA.0)
text(P.CA.0, display = c("rotated"),cex=0.5,col="blue") # TM0
text(P.CA.0, display = c("target"),cex=0.5,col="red") # TOP0
```

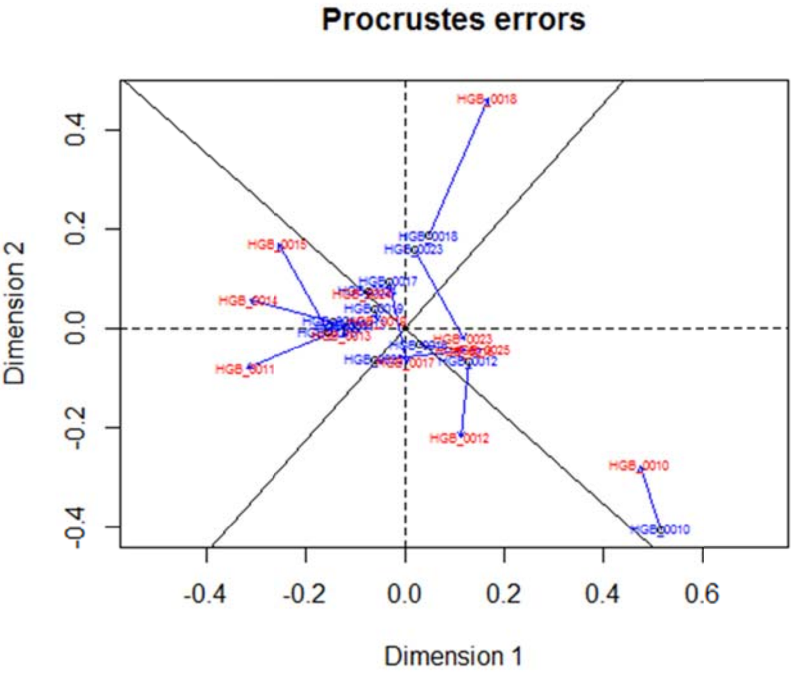

Here we could resolve a bit better the dissimilarities among samples provided by the composition table calculated with MED (TM0). Even if the ordinations were different as indicated by the presence of rather long blue arrows, the two ordination solutions are significantly correlated to each other (r=0.7868, P=0.001).

Therefore the overall relationships between samples seem to be well retrieved using MED or OP.

To determine which OT from the two tables would best match, one can do a correlation analysis of a joined table, as follows:

```
Table_together.0 <- TM0
colnames(Table_together.0)<- paste("MED.",colnames( TM0),sep="")

OP.Table.0<- TOP0
colnames(OP.Table.0)<- paste("OP.",colnames(TOP0),sep="")
Table_together.0 <- cbind(Table_together.0,OP.Table.0)
#as.dist(round(cor(Table_together.0),3))
#plot(hclust(dist(t(Table_together.0))))
require(gplots)
heatmap.2(Table_together.0,col=greenred(10),scale="none",key=TRUE,sy
mkey=FALSE,density.info="none",trace="none",cexRow =0.8)
```

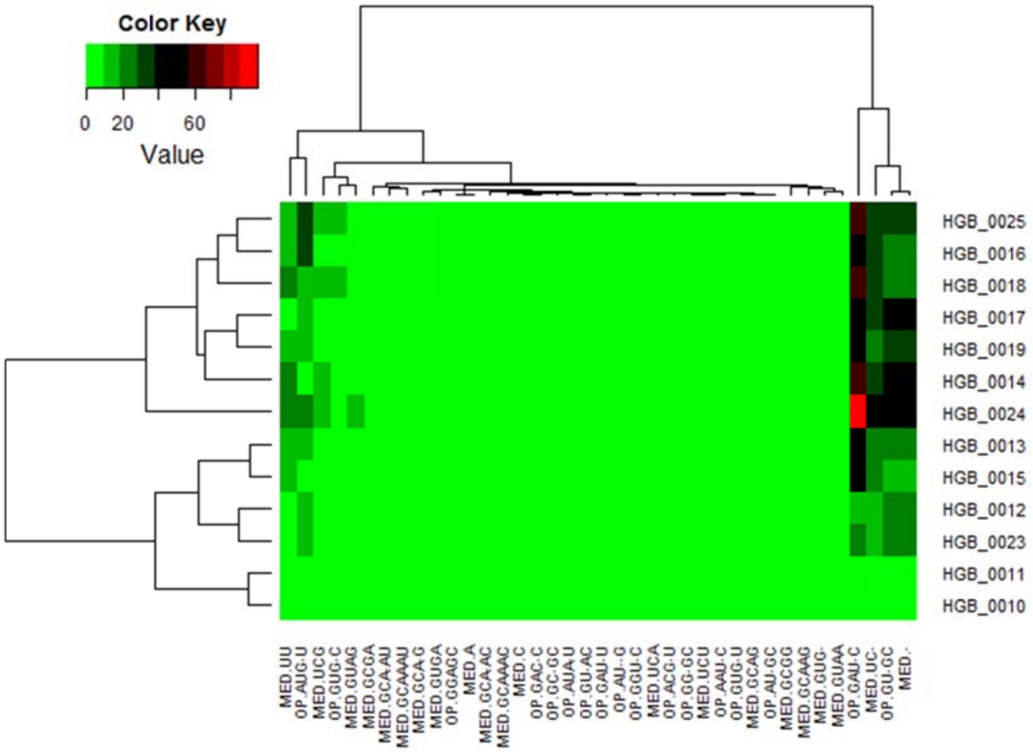


```
MatCorr0 <- as.matrix(as.dist(round(cor(Table_together.0),3)))
#removing some unecessary rows and columns
MatCorr0.1 <-MatCorr0[-grep(pattern = "MED",colnames(MatCorr0)),
                      -grep(pattern = "OP",colnames(MatCorr0))]

# Sorting the correlation table by both OT abundance
TM0.OT.sort <- colSums(TM0[,names(sort(colSums(TM0),decreasing = TRUE))])
  TM0.OT.sort.names <- paste("MED.",names(TM0.OT.sort),sep="")
TOP0.OT.sort <-colSums(TOP0[,names(sort(colSums(TOP0),decreasing =
TRUE))])
  TOP0.OT.sort.names <- paste("OP.",names(TOP0.OT.sort),sep="")

MatCorr0.2 <- MatCorr0.1[TOP0.OT.sort.names,TM0.OT.sort.names]
write.table(MatCorr0.2,"MatCorr0.2.txt",quote=FALSE)# further
rearrangement in Excel
```

Table 1. Correlation table of OT abundances coming from MED or OP.

		OP.GAU-C	OP.GU-GC	OP.AUG-U	OP.GUG-C	OP.GAC-C	OP.GGAGC	OP.GC-GC	OP.GU-AC	OP.GUG-U	OP.AAU-C	OP.ACG-U	OP.AU--G	OP.AU-GC	OP.AUA-U	OP.GAU-U	OP.GG-GC	OP.GGU-C
	Counts	562	348	188	56	4	3	2	2	2	1	1	1	1	1	1	1	1
MED.-	355	**0.864**	**0.999**	0.624	0.556	0.357	-0.027	0.098	-0.117	0.527	0.512	0.284	0.326	0.201	-0.131	-0.193	0.512	0.326
MED.UC-	313	**0.966**	**0.871**	0.716	0.67	0.49	0.108	0.095	-0.148	0.613	0.612	0.108	0.13	0.218	-0.309	-0.002	0.612	0.13
MED.UU	161	**0.913**	0.658	0.356	0.418	0.773	0.3	0.314	0.057	0.34	0.404	-0.222	0.439	0.056	-0.222	0.195	0.404	0.439
MED.UCG	89	**0.891**	**0.829**	0.435	0.631	0.591	0.229	0.216	0.216	0.622	0.669	0.064	0.284	0.174	0.064	-0.157	0.669	0.284
MED.GUAG	60	0.71	0.792	0.752	0.763	0.08	0.125	0.118	0.051	0.584	0.576	0.396	-0.236	0.215	-0.056	-0.326	0.576	-0.236
MED.GCAAAU	29	-0.065	0.044	0.609	0.186	-0.346	-0.035	-0.052	-0.165	0.061	-0.188	-0.188	-0.188	0.271	-0.188	-0.188	-0.188	-0.188
MED.GCGA	28	0.494	0.474	**0.834**	0.521	-0.044	-0.265	-0.3	-0.3	0.698	0.227	-0.142	-0.019	0.719	-0.142	-0.019	0.227	-0.019
MED.GCAAG	27	0.315	0.481	0.281	0.355	-0.037	0.192	0.438	0.284	-0.178	-0.225	0.609	-0.016	-0.016	0.192	-0.016	-0.225	-0.016
MED.GCGG	22	0.44	0.276	0.462	0.725	0.311	0.676	0.34	0.528	0.058	0.294	0.167	-0.216	-0.216	0.039	-0.216	0.294	-0.216
MED.GUAA	18	0.574	0.536	0.684	0.773	0.191	0.366	0.374	0.374	0.541	0.593	-0.087	-0.314	0.14	0.14	-0.314	0.593	-0.314
MED.GUG-	16	0.659	0.631	0.652	**0.912**	0.22	0.324	0.208	0.073	0.749	0.507	0.324	-0.225	0.507	-0.225	-0.225	0.507	-0.225
MED.GCA-AU	15	0.11	0.021	0.714	0.448	-0.256	0.143	0.087	-0.039	0.087	-0.196	-0.196	-0.196	0.313	-0.196	-0.196	-0.196	-0.196
MED.GCA-G	10	0.566	0.52	0.526	0.657	0.286	0.059	-0.102	-0.102	**0.849**	0.317	0.059	0.059	**0.833**	-0.198	0.059	0.317	0.059
MED.GUGA	10	0.154	0.208	0.627	0.509	-0.309	0.059	0.278	0.088	0.469	-0.198	-0.198	-0.198	**0.833**	0.059	-0.198	-0.198	-0.198
MED.GCA-AC	6	0.355	0.343	0.531	0.266	0.03	-0.158	-0.234	-0.234	0.272	-0.158	-0.158	0.527	0.527	-0.158	-0.158	-0.158	0.527
MED.GCAAAC	5	0.49	0.245	0.497	0.245	0.158	-0.228	-0.337	-0.337	0.539	0.365	-0.228	-0.228	0.365	-0.228	0.365	0.365	-0.228
MED.A	4	0.129	-0.103	0.012	0.488	0.359	**0.946**	0.619	**0.879**	-0.16	-0.108	-0.108	-0.108	-0.108	0.243	-0.108	-0.108	-0.108
MED.C	4	0.632	0.341	-0.073	0.23	**1**	0.433	0.178	0.178	0.178	0.433	-0.192	0.433	-0.192	-0.192	0.433	0.433	0.433
MED.GCAG	1	0.172	0.195	0.51	0.463	-0.192	-0.083	-0.123	-0.123	0.677	-0.083	-0.083	-0.083	**1**	-0.083	-0.083	-0.083	-0.083
MED.UCA	1	-0.282	-0.037	0.116	-0.16	-0.192	-0.083	-0.123	-0.123	-0.123	-0.083	-0.083	-0.083	-0.083	-0.083	-0.083	-0.083	-0.083
MED.UCU	1	0.602	0.512	0.247	0.324	0.433	-0.083	-0.123	-0.123	0.677	**1**	-0.083	-0.083	-0.083	-0.083	-0.083	**1**	-0.083

In grey, the Pearson correlation coefficients that were > 0.6 (absolute values). Bold, underlined values are those >0.8. OT names in red color are those which are associated with a correlation coefficient higher than 0.8 at least once.

now filter the OT tables by using the Broken-stick model to only keep OT abundances that are supposed to have occurred not by chance alone.

```
TM.OTAbund <- apply(TM0,2,sum) # overall abundance for each OT
TM.OTAbund_BSM <- Count.BrokenStick(TM.OTAbund,Plot = TRUE)
TM_BSM <- TM0[,TM.OTAbund_BSM$HigherThanBSM]
TM_BSM
          Oligotypes
Samples     - UC- UU
  HGB_0010  0   2  0
  HGB_0011  7   4  1
  HGB_0012 25  15  2
  HGB_0013 22  24 13
  HGB_0014 43  30 25
  HGB_0015 18  24 18
  HGB_0016 28  35 15
  HGB_0017 41  29  6
  HGB_0018 26  29 21
  HGB_0019 35  25 16
  HGB_0023 21  10  6
  HGB_0024 52  52 24
  HGB_0025 37  34 14
TOP.OTAbund <- apply(TOP0,2,sum) # overall abundance for each OT
```

```
TOP.OTAbund_BSM <- Count.BrokenStick(TOP.OTAbund,Plot = TRUE)
TOP_BSM <- TOP0[,TOP.OTAbund_BSM$HigherThanBSM]
TOP_BSM
          Oligotypes
Samples    GAU-C GU-GC AUG-U
  HGB_0010     2     0     3
  HGB_0011     5     7     0
  HGB_0012    19    25    18
  HGB_0013    41    22    11
  HGB_0014    66    42     9
  HGB_0015    45    18     7
  HGB_0016    54    28    30
  HGB_0017    43    41    16
  HGB_0018    61    24    16
  HGB_0019    49    34    15
  HGB_0023    24    20    11
  HGB_0024    95    51    22
  HGB_0025    58    36    30
```

In both cases, only 3 OT were retained, corresponding to:

```
sum(TM_BSM)/sum(TM0)
0.7055319
sum(TOP_BSM)/sum(TOP0)
0.9344681
```

of the total pool of sequences.

Still the total variance of each table is very different:

```
sum(apply(TM_BSM,2,var))
[1] 472.0641
sum(apply(TOP_BSM,2,var))
[1] 953.3205
```

```
mantel(vegdist(TM_BSM), vegdist(TOP_BSM))#Bray-Curtis
Mantel statistic r: 0.9867
      Significance: 0.001

plot.lm.ci1(x=as.numeric(vegdist(TM_BSM)),y=as.numeric(vegdist(TOP_BSM)),m
ain="vegdist (TM_BSM~TOP_BSM)")

mantel(dist(TM_BSM), dist(TOP_BSM))# Euclidean
Mantel statistic r: 0.9754
      Significance: 0.001

plot.lm.ci1(x=as.numeric(dist(TM_BSM)),y=as.numeric(dist(TOP_BSM)),main="d
ist(TM_BSM~TOP_BSM)")
```

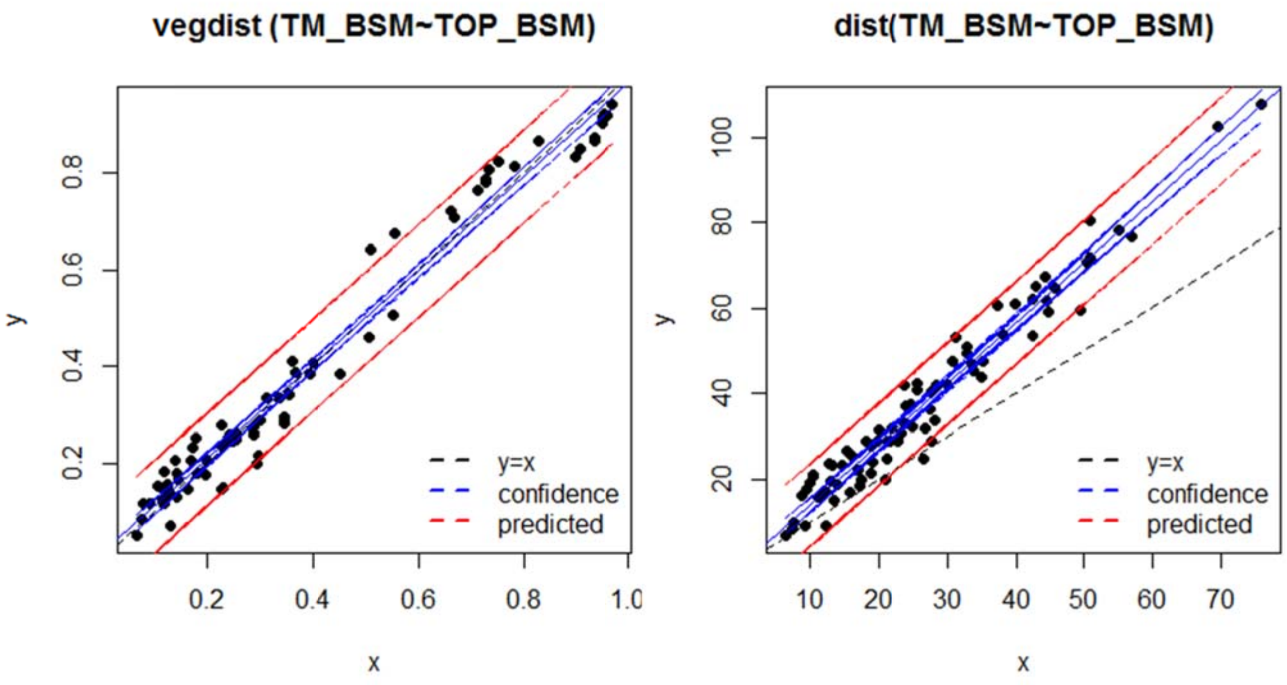

The NMDS based on both filtered dataset had an issue, probably due to the insufficient number of columns. So it is skipped here. When using CA instead of NMDS:

```
CA.TM_BSM <- cca(TM_BSM)
plot(CA.TM_BSM,main="CA.TM_BSM")#,xlim=c(-2,9),ylim=c(-4,2))
CA.TOP_BSM <- cca(TOP_BSM)
plot(CA.TOP_BSM,main="CA.TOP_BSM") #,xlim=c(-3,4))

P.CA_BSM <- protest(CA.TOP_BSM,CA.TM_BSM)
Correlation in a symmetric Procrustes rotation: 0.8793
Significance:  0.002
plot(P.CA_BSM)
text(P.CA_BSM, display = c("rotated"),cex=0.5,col="blue") #TM_BSM
text(P.CA_BSM, display = c("target"),cex=0.5,col="red") #TOP_BSM
```

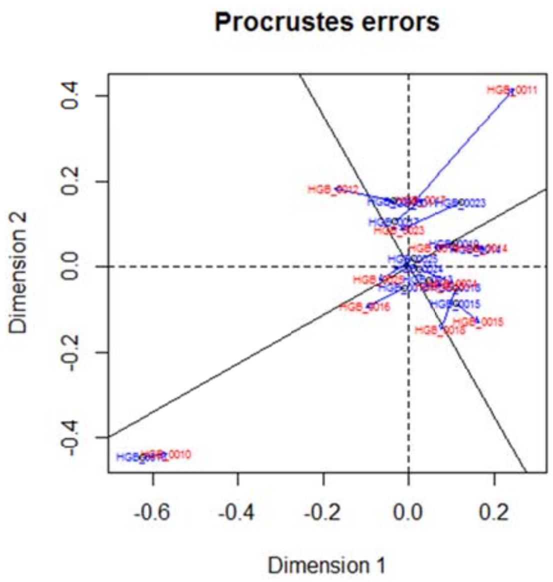

##which OT abundance produced by one technique match other OT in other table?

```
Table_together_BSM <- TM_BSM
colnames(Table_together_BSM)<- paste("MED.",colnames( TM_BSM),sep="")
OP.Table_BSM<- TOP_BSM
colnames(OP.Table_BSM)<- paste("OP.",colnames(TOP_BSM),sep="")
Table_together_BSM <- cbind(Table_together_BSM,OP.Table_BSM)
#as.dist(round(cor(Table_together_BSM),3))
#plot(hclust(dist(t(Table_together_BSM))))
require(gplots)
heatmap.2(Table_together_BSM,col=greenred(10),scale="none",key=TRUE,symkey
=FALSE,density.info="none",trace="none",cexRow =0.8,cexCol=0.7)
```

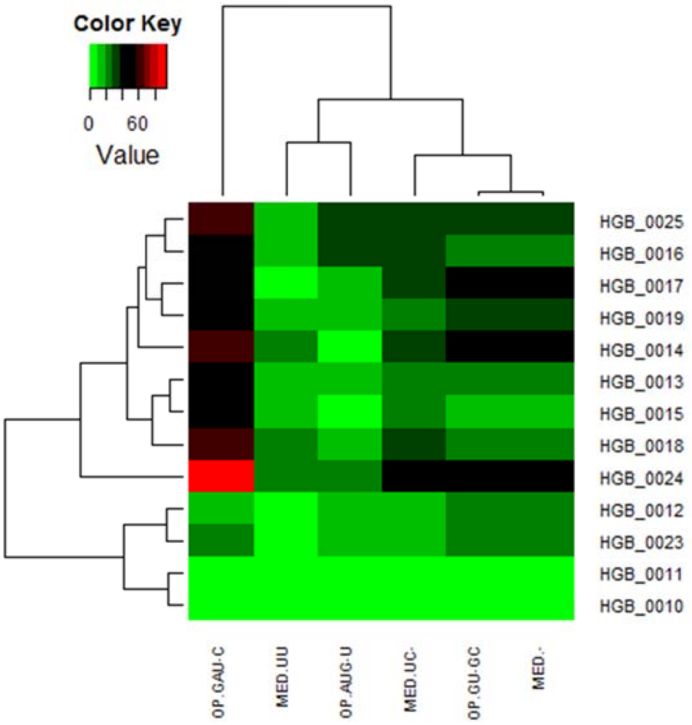


```
MatCorr0_BSM <- as.matrix(as.dist(round(cor(Table_together_BSM),3)))
```

#removing some unnecessary rows and columns

```
MatCorr0.1_BSM <-MatCorr0_BSM[-grep(pattern =
"MED",colnames(MatCorr0_BSM)),
                      -grep(pattern = "OP",colnames(MatCorr0_BSM))]
t(MatCorr0.1_BSM)
        OP.GAU-C OP.GU-GC OP.AUG-U
MED.-      0.864    0.999    0.624
MED.UC-    0.966    0.871    0.716
MED.UU     0.913    0.658    0.356
```

So most of the OT from each approach are well correlated or represented by the other technique.

Summary

Table 2. Summary of the comparison between OP vs. MED, and on using the raw compositional table vs. table filtered after applying the Broken-Stick model.

**Type of data**	**Raw abundance tables**		**Broken-stick model filtering**	
**Method**	**OP**	**MED**	**OP**	**MED**
Table name in the R script	TOP0	TM0	TOP_BSM	TM_BSM
Number of OT	17	21	3	3
Number of singleton OT (%)	8 (47%)	3 (14%)	0 (0%)	0 (0%)
Total variance	974.2	543.0	953.3 (97.9%)[$]	472.1 (86.9%)[$]
RV Coefficient	rv: 0.9848*		rv: 0.9824*	
Mantel test - Bray-Curtis, Euclidean	r: 0.994*, r: 0.981*		r: 0.987*, r: 0.975*	
Correlation of CA ordination plots	r: 0.787*		r: 0.879*	
Number of OT highly correlated (>0.8) to OT produced with the other approach (% to the total number of OT)	11 (64.7%)	10 (47.6%)	2 (66.7%)	3 (100%)

* P<0.01.
[$]percentage referring to the variance in the corresponding raw abundance table.

References

Escouffier, Y. (1973). "Le traitement des variables vectorielles." Biometrics **29**: 751–760.

Legendre, P. and L. Legendre (1998). Numerical Ecology, Elsevier Science B.V., Amsterdam. The Netherlands.
