# Supplementary material for: The R package otu2ot for implementing the entropy decomposition of nucleotide variation in sequence data
Source: Front Microbiol. 2014 Nov 14;5:601. doi: 10.3389/fmicb.2014.00601 (PMC4231947; doi:10.3389/fmicb.2014.00601)
Supplement: Supplementary file 1 [file Presentation1.ZIP › Supplementary Material/Tutorial 4 - One-Pass profiling vs. MED on several FASTA alignments.pdf]

Tutorial 4: One-Pass profiling vs. MED on several FASTA alignments

```
library(otu2ot)
help(package = otu2ot)
```

#all files for each OTU are found in

```
DIR ="E:/Oligotyping/OT.180OTU_fasta/arbTrimmedFASTAsForOligotyping/"
setwd(DIR)
FASTAFILES=dir(DIR)
  #i=76 for "HGB_0013_GXJPMPL01A3OQX.fasta"
NFiles=length(FASTAFILES) #269
```

#1) import all sequences into R

```
Aln.list=vector("list",NFiles) #to store results
names(Aln.list)<- FASTAFILES

system.time(
  for(i in 1: NFiles){
    Aln.list[[i]]<- ImportFastaAlignment(File=FASTAFILES[i]) #path to
FASTA file
  }
)# 82.68 s
```

#2) Decomposing according to the two techniques

```
SEQUENCES=vector("list",NFiles) #to store results
names(SEQUENCES)<-FASTAFILES

for(i in 1: NFiles){
  SEQUENCES[[i]] <- toupper(Aln.list[[i]]$Seqs.only)
}
```

filter across datasets to keep only those with enough entropy

```
system.time(
  EnoughEntropy<- sapply(SEQUENCES,FUN=function(S){
      Check.entropy.nseq(S,minseq=0,entropy.min=0.6)})
) #60.06 s

SEQUENCES.ok <- SEQUENCES[EnoughEntropy]#217 named vector with the file
names
Nok=length(SEQUENCES.ok) # HOw many OK
```

1) use OP

```
LISTOPResults =vector("list",Nok)
names(LISTOPResults)<-names(SEQUENCES.ok)
system.time(
  for(i in 1: Nok){
    LISTOPResults[[i]] <- OnePassProfilingMat(AlignedSequences =
SEQUENCES.ok[[i]],

minseq=21,entropymin=0.6,Plot=FALSE)
  }
)#user 49.53 s
```

2) MED

```
LISTMEDResults = vector("list",Nok)

system.time(
  for(i in 1:Nok){#
        LISTMEDResults[[i]] <- MEDMat(AlignedSequences =
SEQUENCES.ok[[i]],
```

```
minseq=21,entropymin=0.6,Plot=FALSE)
  }
)#several minutes. user 583.99/60 = 9.7 min
length(LISTMEDResults)

names(LISTMEDResults)<-names(SEQUENCES.ok)
```

get the ENV information from the FASTA header.

```
LISTENV=vector("list",Nok) #to store results
system.time(
  for(i in 1:Nok){
    LISTENV[[i]]<-

GetEnvironmentDatafromFileR(File=names(SEQUENCES.ok)[i],Start=2,Stop=9,tes
t=FALSE)
  }
)#6 s
```

#Producing the Sample By OT tables.

```
LIST_TM0=vector("list",Nok) #to store results
names(LIST_TM0)<- names(SEQUENCES.ok)

for(i in 1: Nok){
  LIST_TM0[[i]]<-
    SampleXOT_Table(
      OT.seq.concat=LISTMEDResults[[i]],
      ENV=LISTENV[[i]],
      mosaicPlot=FALSE,
      filterByMinAbund= 0# numeric. minimum abundance for the ot to be
present
    )$SamplexOT.table
}#very fast

LIST_TOP0=vector("list",Nok) #to store results
names(LIST_TOP0)<- names(SEQUENCES.ok)
for(i in 1: Nok){
  if(LISTOPResults[[i]]$OT.freq[1]!=1){
    LIST_TOP0[[i]]<-
      SampleXOT_Table(
        OT.seq.concat=LISTOPResults[[i]][[1]], ################# <
        ENV=LISTENV[[i]],
        mosaicPlot=FALSE,
        filterByMinAbund= 0# numeric. minimum abundance for the ot to be
present
      )$SamplexOT.table
  }
}#
```

#total variance

```
LIST_TM0.var <- sapply(LIST_TM0,FUN=function(S)sum(apply(S,2,var)))
LIST_TOP0.var <- sapply(LIST_TOP0,FUN=function(S)sum(apply(S,2,var)))
```

#RV coefficient

```
LIST_RVcoeff <- rep(NA,Nok)
require(FactoMineR)
for(i in 1:Nok){
  LIST_RVcoeff[i] <-
  coeffRV(LIST_TM0[[i]],LIST_TOP0[[i]])[[1]]
}
summary(LIST_RVcoeff)
Min. 1st Qu.  Median    Mean 3rd Qu.    Max.
 0.7772  0.9576  0.9840  0.9678  0.9960  1.0000
```

#using correspondence analysis to better resolve the finer correspondence between OT abundance and sample

```
require(vegan, quietly =TRUE)
LIST_TOP0.CA <- lapply(LIST_TOP0,FUN=function(S){cca(S)})
LIST_TM0.CA <- lapply(LIST_TM0,FUN=function(S){cca(S)})
```

#Protest can only be used if the two ordination solutions are both 2D. (1D vs. 2D does not work!)

```
CA_1D <- NULL #
for(i in 1: Nok){
  if(LIST_TOP0.CA[[i]]$CA$rank==1){CA_1D=c(CA_1D,i)}
}
for(i in 1: Nok){
  if(LIST_TM0.CA[[i]]$CA$rank==1){CA_1D=c(CA_1D,i)}
}
CA_1D <- unique(CA_1D)
Nok_2D=Nok-length(CA_1D) #198. Nber of 1CD: 19

if(!is.null(CA_1D)){
  LIST_TOP0.CA<-LIST_TOP0.CA[-CA_1D] #removing 1D
  LIST_TM0.CA <- LIST_TM0.CA[-CA_1D]
}

LIST_Protest.CA.r=rep(NA,Nok_2D) #coefficient in a symmetric Procrustes
rotation
LIST_Protest.CA.p=rep(NA,Nok_2D) # its associated P value
system.time(
  for(i in 1:Nok_2D){
    Prot<- protest(LIST_TOP0.CA[[i]],LIST_TM0.CA[[i]])
    LIST_Protest.CA.r[i] <- Prot$scale #coeff
    LIST_Protest.CA.p[i] <- Prot$signif #coeff
  }
)#10.98 s

Protest.table <-
as.data.frame(cbind(Procrustes_r=LIST_Protest.CA.r,P=LIST_Protest.CA.p))
Protest.table.significant <- Protest.table[Protest.table$"P"<0.05,]
Protest.table.significant
nrow(Protest.table.significant)
```

#150 significant, i.e. 150/198 =0.76

```
summary(Protest.table.significant[,1])
   Min. 1st Qu.  Median    Mean 3rd Qu.    Max.
 0.5432  0.7592  0.8825  0.8566  0.9661  1.0000
```

after filtering using Broken-stick model ----------------

#TM0

Which OT in the raw table have abundances higher than predicted by using the Broken-Stick model approach?

```
LIST_TM_BSM=vector("list",Nok) #to store results
names(LIST_TM_BSM)<- names(SEQUENCES.ok)
for(i in 1:Nok){
     TM.OTAbund <- apply(LIST_TM0[[i]],2,sum) # overall abundance for each
OT
     TM.OTAbund_BSM <- Count.BrokenStick(TM.OTAbund,Plot =
FALSE)$HigherThanBSM
     LIST_TM_BSM[[i]] <- LIST_TM0[[i]][,TM.OTAbund_BSM]
}
```

#TOP0

Which OT in the raw table have abundances higher than predicted by using the Broken-Stick model approach?

```
LIST_TOP_BSM=vector("list",Nok) #to store results
names(LIST_TOP_BSM)<- names(SEQUENCES.ok)
for(i in 1:Nok){
  TOP.OTAbund <- apply(LIST_TOP0[[i]],2,sum) # overall abundance for each
OT
```

```
  TOP.OTAbund_BSM <- Count.BrokenStick(TOP.OTAbund,Plot =
FALSE)$HigherThanBSM
  LIST_TOP_BSM[[i]] <- LIST_TOP0[[i]][,TOP.OTAbund_BSM]
}
```

removing the empty slots after BSM filtering

```
LIST_TM_BSM.ncol = lapply(LIST_TM_BSM,ncol) # counting the nber of OT in
each slot
 table(sapply(LIST_TM_BSM.ncol,is.null))
FALSE  TRUE
  149    68
 table(unlist(LIST_TM_BSM.ncol)) #
```

#0 2 3 4 5 6 7 12 # <- ncol

#70 43 18 8 5 2 2 1 # <- occurrence

```
LIST_TM_BSM_ncol_vec <- unlist(LIST_TM_BSM.ncol)
LIST_TM_BSM1 <- LIST_TM_BSM[names(which(LIST_TM_BSM_ncol_vec !=0 &
!is.null(LIST_TM_BSM_ncol_vec) ))]
length(LIST_TM_BSM1)
[1] 79
```

```
LIST_TOP_BSM.ncol = lapply(LIST_TOP_BSM,ncol) # counting the nber of OT in
each slot
table(sapply(LIST_TOP_BSM.ncol,is.null))
FALSE  TRUE
  161    56
 table(unlist(LIST_TOP_BSM.ncol)) #
```

#0 2 3 4 5 6 7 8 9 10 11 12 13 18 25 29 39 # <- ncol

#38 35 25 16 12 7 5 4 3 6 4 1 1 1 1 1 1 # <- occurrence

```
LIST_TOP_BSM_ncol_vec <- unlist(LIST_TOP_BSM.ncol)
LIST_TOP_BSM1 <- LIST_TOP_BSM[names(which(LIST_TOP_BSM_ncol_vec !=0 &
!is.null(LIST_TOP_BSM_ncol_vec) ))]
length(LIST_TOP_BSM1)
[1] 123
```

Conclusion: More OP than TM that are finally available

need to find the common set of data

```
DataNamesInCommon <- intersect(names(LIST_TM_BSM1),names(LIST_TOP_BSM1))

Ncomm=length(DataNamesInCommon)#67 only in common
LIST_TM_BSM1comm<- LIST_TM_BSM1[DataNamesInCommon]
LIST_TOP_BSM1comm <- LIST_TOP_BSM1[DataNamesInCommon]
```

#total variance

```
LIST_TM_BSM1comm.var <-
sapply(LIST_TM_BSM1comm,FUN=function(S)sum(apply(S,2,var)))
LIST_TOP_BSM1comm.var <-
sapply(LIST_TOP_BSM1comm,FUN=function(S)sum(apply(S,2,var)))

Variance_TM_TOP.BSM <-
as.data.frame(cbind(TM_BSM=LIST_TM_BSM1comm.var,TOP_BSM=LIST_TOP_BSM1comm.
var))
  plot.lm.ci(Variance_TM_TOP.BSM[,2],Variance_TM_TOP.BSM[,1],
             main="Variance: TM [y]  TOP [x] (BSM)",
             xlim=c(0,950),ylim=c(0,950)
             )
```

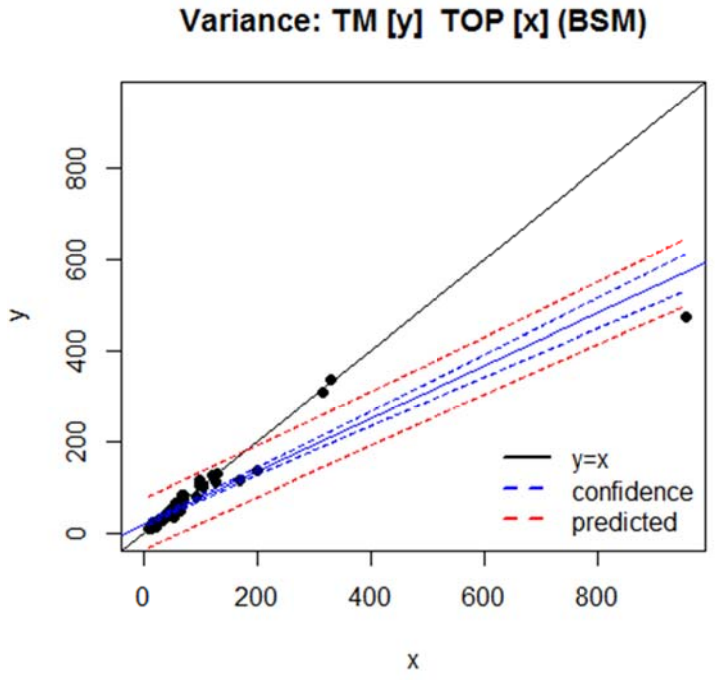


```
 plot.lm.ci(Variance_TM_TOP.BSM[,2],Variance_TM_TOP.BSM[,1],
           main="Variance: TM [y]  TOP [x] (BSM)",
           xlim=c(0,400),ylim=c(0,400)
  )
```

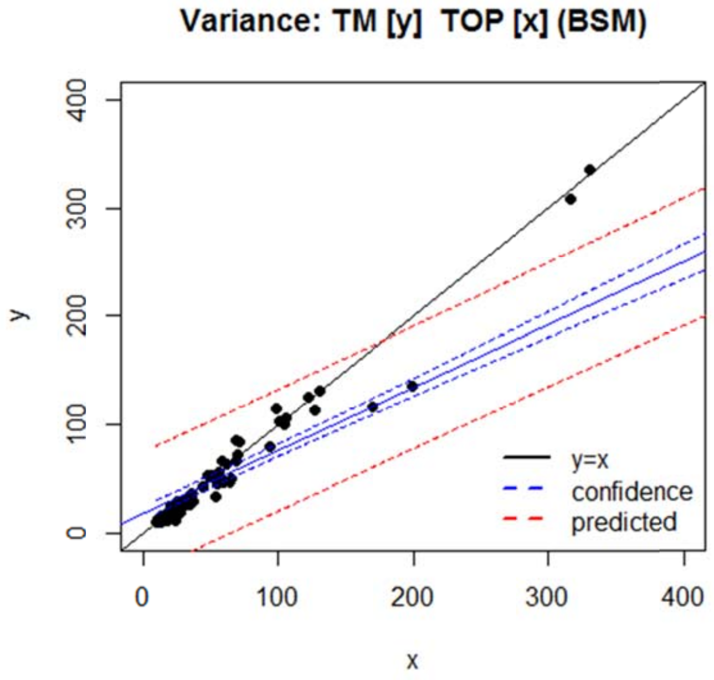

##Conclusions after: most of the points are on the 1:1 line
weird points:

```
  Weird <- subset(x = Variance_TM_TOP.BSM,subset = TOP_BSM>150 &
TOP_BSM<210)
  Weird=rbind(Weird,
          subset(x = Variance_TM_TOP.BSM,subset = TOP_BSM>400)
  )
  Weird
  #HGB_0024_HIULTSN01CPGLX.fasta 116.0769 169.6538
```

```
#HGB_0025_HDPDD3401D6L31.fasta 136.3077 199.3590
#HGB_0013_GXJPMPL01A3OQX.fasta 472.0641 953.3205
```

##removing 3 points

```
Variance_TM_TOP.BSM_ <-
Variance_TM_TOP.BSM[setdiff(rownames(Variance_TM_TOP.BSM),rownames(Weird))
,]
plot.lm.ci(Variance_TM_TOP.BSM_[,2],Variance_TM_TOP.BSM_[,1],
           main="Variance: TM [y]  TOP [x] (BSM)",
           xlim=c(0,150),ylim=c(0,150)
)
```

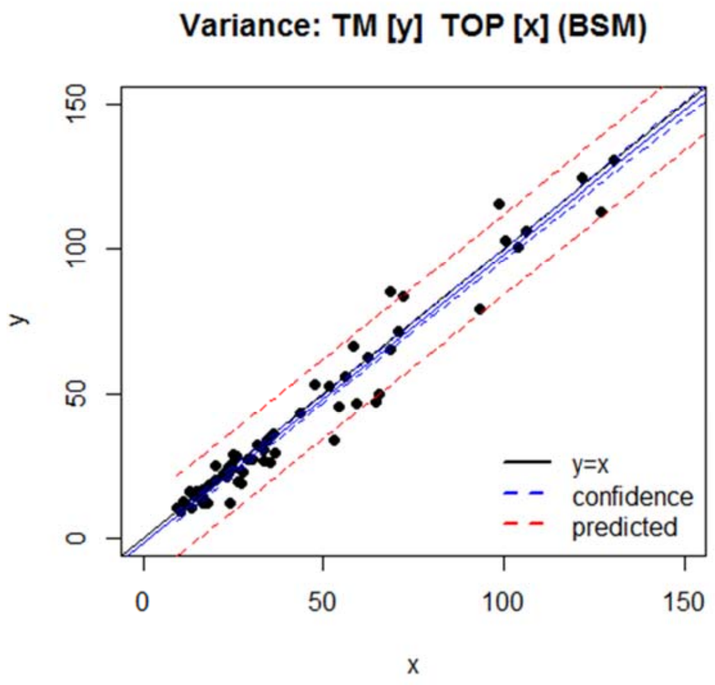

#link with max(entropy) or number of components?

```
NberComponent <- function(MatSeq,entropy.min=0.6){
    return(length(which(apply(MatSeq,2,CalcEntropy.seq)>entropy.min)))
}
LIST.NberComp <- sapply(SEQUENCES.ok,NberComponent)
LIST.NberComp [rownames(Weird)]
```

#HGB_0024_HIULTSN01CPGLX.fasta HGB_0025_HDPDD3401D6L31.fasta HGB_0013_GXJPMPL01A3OQX.fasta

#10 1 5

```
barplot(LIST.NberComp,names.arg="",,main="Nber of components per
dataset")
abline(h=c(1,5,10),col="red",lty=2)
```

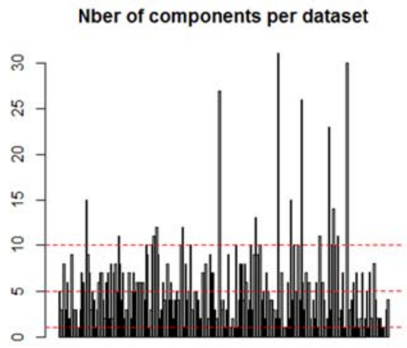

```
summary(LIST.NberComp)
 Min. 1st Qu.  Median    Mean 3rd Qu.    Max.
 1.000   2.000   4.000   5.253   7.000  31.000
```

#Conclusions not the nber of components

```
MaxEntropy <- function(MatSeq){
  return(      max(apply(MatSeq,2,CalcEntropy.seq))      )
}
LIST.MaxEntropy <- sapply(SEQUENCES.ok,MaxEntropy)
LIST.MaxEntropy [rownames(Weird)]
```

#HGB_0024_HIULTSN01CPGLX.fasta HGB_0025_HDPDD3401D6L31.fasta HGB_0013_GXJPMPL01A3OQX.fasta

#1.3207916 0.9403435 1.5584605

```
barplot(LIST.MaxEntropy,names.arg="",main="Max entropy per dataset")
abline(h=as.numeric(LIST.MaxEntropy [rownames(Weird)]),col="red",lty=2)
```

#Conclusions not the max entropy

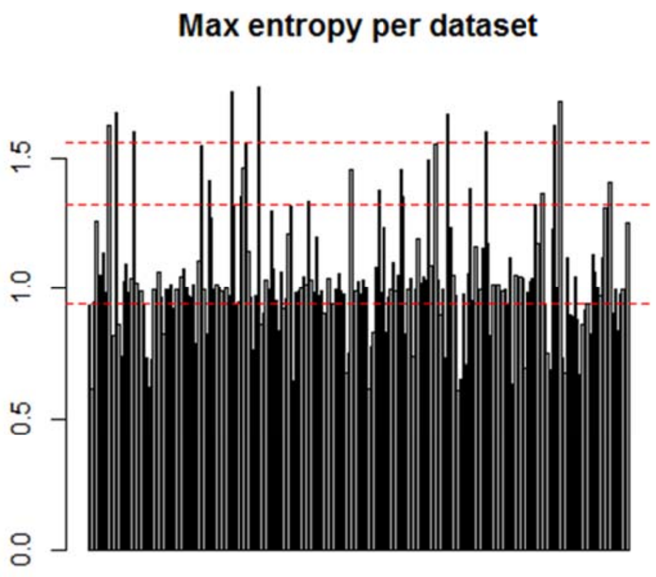


```
par(pch=16)
plot(LIST.MaxEntropy,LIST.NberComp,xlab="Max entropy",ylab="Number of components >0.6")

points(LIST.MaxEntropy[rownames(Weird)],LIST.NberComp[rownames(Weird)],col="red")
```

in red points, the 3 datasets not falling on the straight line

```
lines(lowess(LIST.MaxEntropy,LIST.NberComp),lty=2,lwd=2)
```

conclusion: cannot find the reason. Also the relationship between max entropy and nber of components is not predictable

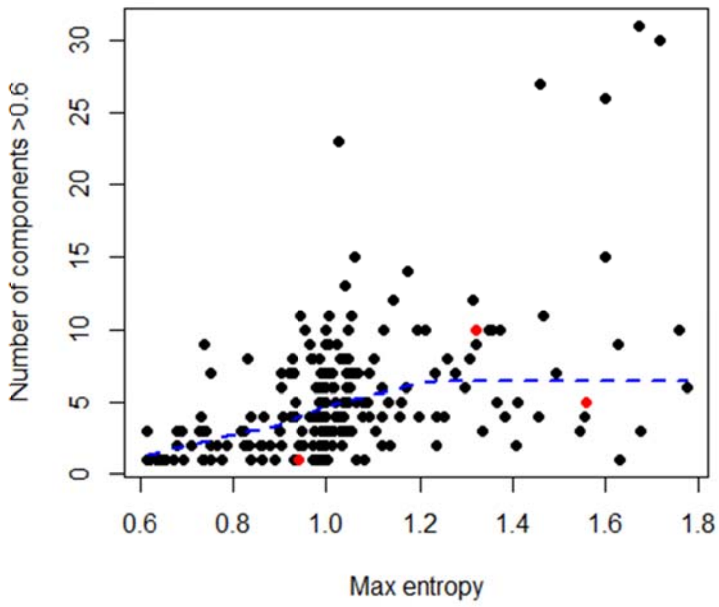

high variance == high entropy?

```
rownames(Variance_TM_TOP.BSM_ )
plot(LIST.MaxEntropy[rownames(Variance_TM_TOP.BSM_ )],
     Variance_TM_TOP.BSM_ [,1],xlab="Max entropy",ylab="Variance")
```

conclusion: no relationships

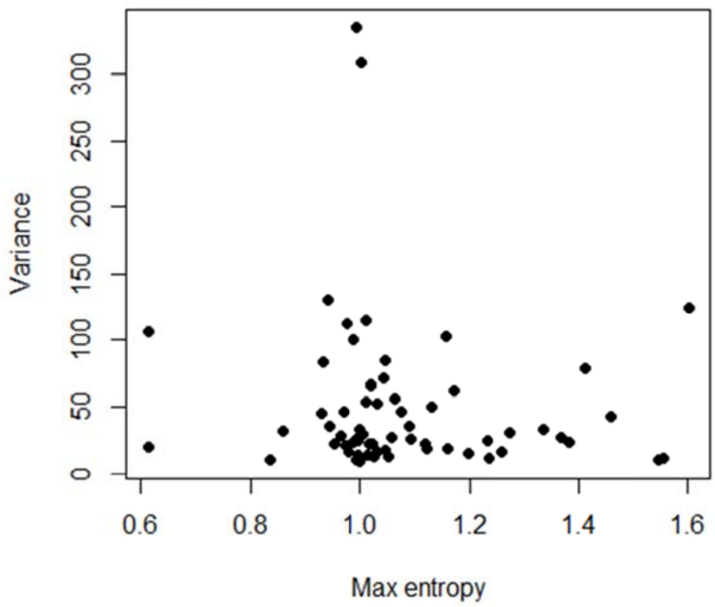

high variance == large nber of components?

```
plot(LIST.NberComp[rownames(Variance_TM_TOP.BSM_ )],
   Variance_TM_TOP.BSM_ [,1],
```

```
    xlab="Number of components",ylab="Variance")
  cor.test(LIST.NberComp[rownames(Variance_TM_TOP.BSM_ )],
      Variance_TM_TOP.BSM_ [,1])#t = -0.8342, df = 62, p-value = 0.4074
```

conclusion: no relationships

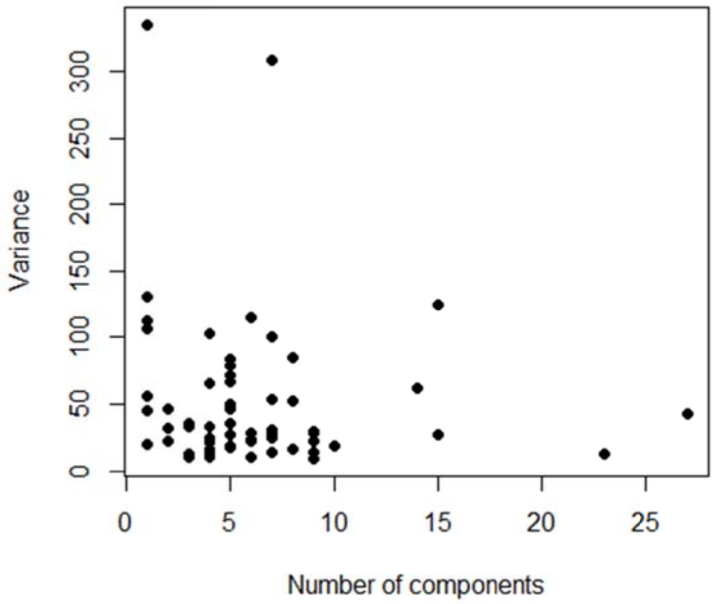

#RV coefficient

```
LIST_RVcoeff_BSM1comm <- rep(NA,Ncomm)
require(FactoMineR)
for(i in 1:Ncomm){
  LIST_RVcoeff_BSM1comm[i] <-
    coeffRV(LIST_TM_BSM1comm[[i]],LIST_TOP_BSM1comm[[i]])[[1]]
}
summary(LIST_RVcoeff_BSM1comm)
```

#Min. 1st Qu. Median   Mean 3rd Qu.   Max.

#0.7920 0.9403 0.9646 0.9537 0.9883 1.0000

#using correspondence analysis to better resolve the finer correspondence between OT abundance and sample

```
require(vegan)
#issue with empty rows
Names_OK_TOP_CA <-
names(which(sapply(LIST_TOP_BSM1comm,FUN=function(S){any(rowSums(S)==0)})=
=FALSE))
Names_OK_TM_CA <-
names(which(sapply(LIST_TM_BSM1comm,FUN=function(S){any(rowSums(S)==0)})==
FALSE))

Names_OK_both_CA <- intersect(Names_OK_TOP_CA,Names_OK_TM_CA)#57
Ncom1 <- length(Names_OK_both_CA)

LIST_TOP_BSM1comm.CA <-
lapply(LIST_TOP_BSM1comm[Names_OK_both_CA],FUN=function(S){cca(S)})
LIST_TM_BSM1comm.CA <-
lapply(LIST_TM_BSM1comm[Names_OK_both_CA],FUN=function(S){cca(S)})
```

#Protest can only be used if the two ordination solutions are both 2D. (1D vs. 2D does not work!)

```
CA_1D1com1 <- NULL #
for(i in 1: Ncom1){
  if(LIST_TOP_BSM1comm.CA[[i]]$CA$rank==1){CA_1D1com1=c(CA_1D1com1,i)}
```

```
}
for(i in 1: Ncom1){
  if(LIST_TM_BSM1comm.CA[[i]]$CA$rank==1){CA_1D1com1=c(CA_1D1com1,i)}
}
CA_1D1com1 <- unique(CA_1D1com1)
Nok_2D1com1=Ncom1-length(CA_1D1com1) #25 (48%). 32 in CA_1D1com1
```
#Conclusions: already half have a very different representation (2D vs. 1D)

```
if(!is.null(CA_1D1com1)){
  LIST_TOP_BSM1comm2.CA<-LIST_TOP_BSM1comm.CA[-CA_1D1com1] #removing 1D
  LIST_TM_BSM1comm2.CA <- LIST_TM_BSM1comm.CA[-CA_1D1com1]
}

LIST_Protest.CA_BSM1comm.r=rep(NA,Nok_2D1com1) #coefficient in a symmetric
Procrustes rotation
LIST_Protest.CA_BSM1comm.p=rep(NA,Nok_2D1com1) # its associated P value
for(i in 1:Nok_2D1com1){
  Prot1comm<-
protest(LIST_TOP_BSM1comm2.CA[[i]],LIST_TM_BSM1comm2.CA[[i]])
  LIST_Protest.CA_BSM1comm.r[i] <- Prot1comm$scale #coeff
  LIST_Protest.CA_BSM1comm.p[i] <- Prot1comm$signif #coeff
}

Protest.table_BSM1comm <-
as.data.frame(cbind(Procrustes_r=LIST_Protest.CA_BSM1comm.r,

P=LIST_Protest.CA_BSM1comm.p))
nrow(Protest.table_BSM1comm)#25
Protest.table.significant_BSM1comm <-
Protest.table_BSM1comm[Protest.table_BSM1comm$"P"<0.05,]
Protest.table.significant_BSM1comm
nrow(Protest.table.significant_BSM1comm)#19 significant, i.e. 19/25 =0.76
nrow(subset(Protest.table.significant_BSM1comm,Procrustes_r>0.8)) # 7/25 =
0.28
summary(Protest.table.significant_BSM1comm[,1])
```
Min. 1st Qu. Median Mean 3rd Qu. Max.

0.5706 0.6487 0.7089 0.7707 0.9183 1.0000
