# Supplementary material for: The R package otu2ot for implementing the entropy decomposition of nucleotide variation in sequence data
Source: Front Microbiol. 2014 Nov 14;5:601. doi: 10.3389/fmicb.2014.00601 (PMC4231947; doi:10.3389/fmicb.2014.00601)
Supplement: Supplementary file 1 [file Presentation1.ZIP › Supplementary Material/otu2ot_1.4.zip!/otu2ot/html/00Index.html]

R: Calculate oligotypes for aligned FASTA sequences

# Calculate oligotypes for aligned FASTA sequences

---

## Documentation for package ‘otu2ot’ version 1.4

- DESCRIPTION file.

## Help Pages

|  |  |
| --- | --- |
| otu2ot-package | The package implements the oligotyping procedure in R. |
| CalcEntropy.seq | calculates the entropy for a nucleotide sequence provided as a vector of bases |
| CalcEntropy.word | calculates the entropy for a concatenated nucleotide sequence |
| Check.entropy.nseq | Check for a subset of FASTA sequences provided as a list, whether some minimum requirements are met |
| Count.BrokenStick | Applies the Broken-Stick model to a vector of count data. |
| GetEnvironmentDatafromFileR | Parse the FASTA headers to retrieve the sample information. |
| HGB\_0013.fasta | FASTA alignment corresponding to OTU "HGB\_0013\_GXJPMPL01A3OQX" |
| ImportFastaAlignment | Import a FASTA alignment from a file |
| MED | Minimum Entropy Decomposition |
| MEDMat | Minimum Entropy Decomposition |
| mock.fasta | mock data for testing the oligotyping pipeline. |
| OnePassProfiling | Provides oligotyping based on a one-pass entropy profiling, without subsequent minimum entropy decomposition. |
| OnePassProfilingMat | Provides oligotyping based on a one-pass entropy profiling, without subsequent minimum entropy decomposition. |
| otu2ot | The package implements the oligotyping procedure in R. |
| plot.lm.ci | plots a x,y plot with the result of a linear regression model (y ~x), with its confidence and prediction bands. |
| plotEntropy | Plot the entropy profile for a matrix of sequences |
| SampleXOT\_Table | creates a Sample-by-OT table. |
